# Retrospective analysis of genetic diversity, clinical manifestations and risk factors, during outbreak of lumpy skin disease virus

**DOI:** 10.1371/journal.pone.0358559

**Published:** 2026-10-16

**Authors:** Mudassar Mohiuddin, Hira Hameed, Muhammad Waqar Aziz, Tayyaba Asghar, Yehia Hazzazi, Mohsen A. Khormi, Layla A. Almutairi, Essam H. Ibrahim, Mudasser Habib, Muhammad Taslim Ghori, Muhammad Salahuddin Shah

**Affiliations:** 1 Department of Microbiology, Faculty of Veterinary and Animal Sciences, The Islamia University of Bahawalpur, Bahawalpur, Pakistan; 2 Department of Microbiology, The Government Sadiq College Women University, Bahawalpur, Pakistan; 3 Provincial Diagnostic Laboratory, Livestock and Dairy Development Department, Lahore, Pakistan; 4 Department of Biosciences, COMSATS University Islamabad, Sahiwal, Pakistan; 5 Department of Biology, College of Science, Jazan University, Jazan, Saudi Arabia; 6 Department of Biology, College of Science, Princess Nourah bint Abdulrahman University, Riyadh, Saudi Arabia; 7 Biology Department, Faculty of Science, King Khalid University, Abha, Saudi Arabia; 8 Blood Products Quality Control and Research Department, National Organization for Research and Control of Biologicals, Cairo, Egypt; 9 Nuclear Institute for Agriculture and Biology College, Pakistan Institute of Engineering and Applied Sciences (NIAB-C, PIEAS), Faisalabad, Pakistan; 10 Department of Clinical Medicine and Surgery, Faculty of Veterinary and Animal Sciences, The Islamia University of Bahawalpur, Bahawalpur, Pakistan; CEA, FRANCE

## Abstract

Lumpy skin is a highly host-specific, contagious, transboundary, notifiable disease affecting cattle (*Bos taurus* and *Bos indicus*) and water buffalo (*Bubalus bubalis*), and also reported in some wild ruminants. The spread and reporting of Lumpy skin disease (LSD) in the past five years in disease-free countries, mainly in Pakistan, has highlighted the threat of its emergence and re-emergence in neighboring areas. Notably significant outbreaks of this disease have been reported in the neighboring countries of Pakistan during the last decades and are thought to be the reason for the first reported outbreak of LSD in Pakistan in the year 2021−22. This study targeted seven locations and collected samples and clinical data of LSD-suspected animals, focusing on clinical presentation, farm-level risk factors, and molecular characterization via PCR and subsequent phylogenetic relatedness of sequenced isolates. Out of the collected samples (n = 285), 117 cases were found positive for LSDV. The most frequent clinical signs included fever (83.7%), skin nodules (94.8%), emaciation (88.0%), and snoring (83.7%), all of which were statistically significant (p ≤ 0.05). Significant farm-level risk factors associated with LSDV positive cases included the use of shared water sources (64.9%), treatment by quack practitioners (79.4%), improper handling of manure (22.2%), and lack of disinfection (15.3%), with significant *p-values* ≤0.05. Molecular confirmation of LSDV was performed by PCR amplification of a 554-bp fragment of the RPO30 gene, a commonly used molecular marker for LSDV detection and characterization. The sequenced isolates showed close genetic similarity to strains reported from India, Bangladesh, and China, indicating a regional genetic relatedness among circulating LSDV strains. The study highlights that enhanced biosecurity, farmer education, vaccination, and veterinary guidance can prevent disease spread, improve livestock health, and reduce economic losses.

## 1 Introduction

Lumpy skin disease is caused by a 320 x 260 nm enveloped double-stranded DNA virus known as Lumpy skin disease virus (LSDV), which belongs to the genus Capripoxvirus and family Poxviridae [1–3]. It is a contagious disease characterized by fever [4], enlargement of superficial lymph nodes, and round, elevated, and dense nodules on the skin that later transform into open wounds and often lead to secondary bacterial infections that result in death in some animals [5,6]. Cattle and water buffaloes are primarily affected by this virus. The morbidity and mortality rates fluctuate between 10–15% and 1–5%, respectively [7]. These variations are likely due to genetic differences among breeds, viral virulence, and environmental fluctuations [8]. The disease causes significant economic losses through trade restrictions, financial breakdowns in the livestock industry, and decreases animal production, including low milk yield and other animal products [9]. The average treatment cost per animal is approximately PKR 6869.69 [10]. The total economic loss is estimated to be approximately five million USD during an outbreak [11]. Since arthropods do not allow the virus to multiply or circulate, it is hypothesized that LSDV is mechanically transmitted through these vectors. The remarkable environmental stability of the virus allows it to persist in a wide variety of vectors [12]. The rate of mechanical viral transmission is negatively correlated with the virus survival time between vector blood feed [13]. Additional significant risk factors include the existence of water bodies, the spread of the disease under different agro-climatic conditions, and the introduction of new animals to the herd [14].

Lumpy skin disease (LSD) outbreaks have occurred in various Asian countries, including Bangladesh, India, China, Nepal, Bhutan, Vietnam, Myanmar, Sri Lanka, and Thailand [15]. The disease was first reported in Zambia in 1929 [16,17]. Between 1943 and 1977, the disease spread to North Sudan, Nigeria, Mauritania, Mali, Ghana, and Liberia [18]. From 1929 to 1986, the deadly disease was limited to sub-Saharan African nations [19]. LSD was officially recognized in Egypt’s Suez Governorate in 1988 and spread to Israel in 1989 [20,21]. Several outbreaks have been recorded in Middle Eastern nations after 2000, and LSD is now considered a common disease in these areas [21]. LSD outbreaks occurred in several areas of Ethiopia, including Amhara and Oromiya in 2000/2001, Oromiya and SNNP in 2003/2004, and Tigray, Amhara, and Benishangul in 2006/2007 [22]. By the end of 2013, the disease had spread to Turkey and Iraq, and to the Russian Federation in 2015 [23]. LSD was detected in China and India in 2019 [24]. Pakistan was affected for the first time in its history by the end of 2021 [25,26]. In Bhakkar and Jhang, the prevalence of the LSD virus was found to be 38.75% and 33.75%, respectively [27]. Seroprevalence of LSD reported in district Jhelum was 20.5%, Rawalpindi 19.5%, Attock 18%, and Chakwal 19.5% [28]. It is now believed that the disease spread across the country after the report of the first case from the Cholistan and Thar desert area [29]. Southern Punjab was the most severely affected area, having the highest frequency of Lumpy skin disease cases reported [30–33].

Dairy production, which relies heavily on livestock, is a major component of Pakistan’s economy [34]. Unfortunately, recurrent LSD outbreaks have had a significant negative impact on the cattle industry, resulting in the deaths of a large number of cattle and, to a lesser extent, buffaloes [35]. In addition to being fatal, the virus reduces cattle productivity, resulting in significant economic loss. Molecular diagnostic methods such as PCR-based amplification of reference genes and genetic relatedness are well documented for LSDV detection in Pakistan and neighboring countries, including China, India, Bangladesh, Nepal, and Egypt. Simultaneously, epidemiological investigations in these countries have highlighted the importance of farm-level risk factors. Thus, this study aimed to identify the specific LSDV strains responsible for these losses. In the community, epidemiological and molecular identification can be used to treat and control lumpy skin disease. The combined approach of this study will contribute to the development of more effective control strategies, preventing future outbreaks and reducing their impact on the cattle industry and national economy.

## 2 Materials and methods

### 2.1 Attributes of the study area

The investigated area comprises the region where Lumpy skin disease was first reported in Pakistan. This area is popularly known as the Cholistan desert (Rohi in the local dialect). This desert is also connected to the Thar Desert, extending into the Sindh province of Pakistan and the neighboring country of India. Greater Cholistan is a largely sandy territory in the desert’s south and west, up to the border with India. The human population in Cholistan is approximately 155,000, while the livestock population reaches 1,318,000. The animal health care facilities are very rare and are available to livestock owners in the form of mobile clinics and temporary dispensaries. The samples and clinical data for this study were taken from seven locations in the outbreak area, namely: Khanpur, Bahawalpur, Khairpur Tamewali, Chishtian, Bahawalnagar, Yazman, and Fort Abbas.

### 2.2 Ethical approval and consent

The study protocols were reviewed and approved by the Institutional Review Board (or Ethics Committee) of The Islamia University of Bahawalpur (MICRO-1/2022/398). The ethical approval covered both animal sampling procedures and questionnaire-based interviews with livestock owners. Verbal informed consent (S1 File) was obtained and documented before participation, and no personally identifiable information was recorded. Animal handling, sampling, and reporting procedures were performed in strict accordance with ARRIVE guidelines and applicable national and local regulations governing animal research and welfare. Interviews were conducted by trained veterinary personnel familiar with field epidemiology. To minimize recall bias, owners were interviewed during or shortly after active outbreaks, and responses were cross-verified with clinical manifestations.

### 2.3 Study design, area, and Sampling strategy

The outbreaks reported during the months from January to September 2022, in Cholistan and surrounding areas along the border, were observed for the collection of clinical data as well as samples (Table 1). A total of 29 farms were included in this study, with herd size ranging from 18 to 43 animals per farm. Farms were selected by a specifically guided approach of convenience sampling, with an emphasis on places, where outbreaks were reported to the local veterinary authorities or where animals with similar clinical signs and symptoms were determined upon field visit. A total of 285 cattle were sampled from these farms. Among them, from 207 animals, skin scab samples were collected, while from 78 animals, blood samples were collected, based on owner consent and sampling feasibility.

**Table 1:** Specimen, number, and breed history of collected samples.

Time of collection	Locations	Cattle Breed	No. of Farms	No. of animals sampled	Scab samples (n)	Blood samples (n)
January 2022	Khanpur	Cross	4	44	34	10
March 2022	Bahawalpur	Cross/ Indigenous	4	49	37	12
April 2022	Khairpur Tamewali	Cross/ Indigenous	6	55	42	13
May 2022	Chishtian	Cross/ Indigenous	7	68	39	29
July 2022	Bahawalnagar	Indigenous	3	38	31	07
August, September 2022	Fort Abbas	Cross	5	31	24	07
**Total**			**29**	**285**	**207**	**78**

### 2.4 Inclusion and exclusion criteria

Cattle presented with characteristic clinical manifestations of LSD including fever, generalized or localized cutaneous nodules, enlarged superficial lymph nodes, limb or brisket edema, and nasal or ocular discharge, were included in the present study. While clinically sick animals without characteristic LSD lesions (generalized or localized cutaneous nodules) and without lymph nodes enlargement were excluded from the study. Cases with non-specific clinical signs not compatible with LSD were also not considered for sampling. Skin scabs samples were collected from animals with active disease, while blood samples were obtained from animals in the recovery phase; each sample represented a single, distinct animal.

### 2.5 Data collection and risk factor assessment

The clinical and epidemiological data were obtained using a structured questionnaire (S2 File) completed during face-to-face interviews with animal owners or primary handlers. The questionnaire included data on possible risk factors, such as animal movement, contact with infected animals, presence of vectors, water sources, manure management practices, treatment history, fiction beliefs, and knowledge of LSD among the owners.

### 2.6 Sample collection and handling

Blood samples were collected in sterile tubes containing an anticoagulant (EDTA) using disposable syringes under aseptic conditions and were properly labeled. Samples (scabs and blood) were kept in zip-lock polythene bags labeled with the animal’s location, household ID, and species. The samples were kept in an insulated cooler at 4°C until they were transported to the laboratory.

### 2.7 DNA Extraction and molecular confirmation of LSDV

Skin scabs and blood samples were processed separately for DNA extraction and PCR analysis. Initially, the skin scab samples were prepared for DNA extraction using a manual method. Briefly, skin scabs were cut into small pieces using a sterile scalpel, and approximately 400–500 mg of sample was added to 500 µl of sterile PBS (pH 7.4) and homogenized. DNA extraction was performed for tissue homogenates (homogenized skin samples) and whole blood using the commercially available QIAamp DNA Mini Kit (Qiagen, USA).

For molecular confirmation, lumpy skin viral DNA was confirmed via amplifying the 554 bp region in the RPO30 gene using primers; forward primer: 5’-CAGCTGTTTGTTTACATTTGATTTTT-3’ and reverse primer: 5’-TCGTATAGAAACAAGCCTTTAATAGA-3’ [36]. The RPO30 gene was chosen for having high discriminatory potential in the genus Capripoxvirus and reliably distinguishes between LSDV and sheep pox virus (SPPV) as well as goat pox virus (GTPV). The reaction mixture used for PCR was 20ul; 0.8 µL Taq polymerase, 2.5 µL dNTPs, 2 µL PCR buffer (NH_4_)_2_SO_4_, 2.5 µL of MgCl_2_, 0.8 µL of each primer, 1.5 µL template DNA, and 9.1 µL distilled H_2_O. PCR was performed in a thermocycler (Bio-Rad, UK) under amplifying conditions; initial denaturation cycle at 95°C for 4 min, followed by 40 cycles of denaturation, annealing, and extension at 95°C for 30s, 55°C for 30s, and 72°C for 45s, respectively. The final extension step was performed at 72°C for 7 min. The PCR products were observed on a 1.5% gel with a band size of 554 bp. Gel was visualized using ViSafe Red Gel Stain (Vivantis, Malaysia) in the Gel Documentation System (Bio-Rad, USA). A previously confirmed LSDV-positive DNA sample, validated via PCR and maintained in the laboratory, was used as a positive control.

### 2.8 DNA sequencing and phylogenetic analysis

A total of seven representative PCR-positive LSDV isolates from different outbreak locations were selected for partial 554-bp fragment of the RPO30 gene sequencing using Sanger sequencing method (Macrogen, Korea). Sequence chromatograms were manually inspected, and quality checked before phylogenetic analysis. The raw bidirectional Sanger reads of partial 554-bp fragment of the RPO30 gene were edited and trimmed using Bioedit software (Ibis Biosciences, USA). The sequences were submitted to GenBank and are available in the database with accession numbers: OR745275 25/IUB/PK, OR745276 L4/IUB/PK, OR745277 L2/IUB/PK, OR745278 P7/IUB/PK, OR745279 MB/IUB/PK, and OR745280 MI/IUB/PK. These sequences were then compared with already reported sequences in the GenBank for the similarity index (NCBI BLAST). Phylogenetic analysis was performed using MEGA-10 software. The resulting partial RPO30 LSDV sequences were aligned using ClustalW in the BioEdit program [20]. The aligned sequences were then compared with previously reported sequences deposited in GenBank using BLAST software at NCBI (https://www.ncbi.nlm.nih.gov/). The maximum neighbor-joining method (MEGA v.11) was employed to construct a phylogenetic tree with a Bootstrap value set at 1000 replications to estimate confidence in the branching patterns of the trees. RPO30 sequences of GTPV and SPPV were used as outgroups for the phylogenetic analysis.

### 2.9 Statistical analysis

Data were summarized as frequencies (n) and percentages with 95% confidence intervals (CI). Associations between infection status (LSDV positive/negative) and categorical variables, including clinical signs and risk factors, were assessed using the chi-square (χ^2^) test [37]. For tables with small expected cell counts, the χ^2^ test was performed with Monte Carlo simulation (10,000 replicates) to obtain exact *p*-values. Pearson’s correlation coefficients (r) were calculated to determine the relationships between different clinical signs and farm-level risk factors. Statistical significance was determined at a *p-value* of <0.05. All statistical analyses were performed using SPSS version 20.0 (SPSS Inc., Chicago, IL, USA). Due to the exploratory nature of the analysis and the use of predefined categorical variables, formal correction for multiple comparisons (e.g., Bonferroni adjustment) was not applied.

## 3 Results

### 3.1 Descriptive epidemiology

Lumpy skin disease (LSD) is a trans-boundary viral infection primarily affecting cattle (indigenous/crossbred). Younger animals, particularly dairy cattle and calves, were more commonly infected. Clinical disease can appear in various forms, ranging from minor to severe. During the first outbreak in Pakistan, the clinical signs were not very severe. The LSD has a low mortality rate but causes debilitation and poor health in cattle for months after infection. Based on the virulence of the strains and the susceptibility of the cow breed, the clinical manifestations of LSD might range from severe to mild or sometimes asymptomatic.

### 3.2 Clinical manifestations

There were several clinical signs, including fever, emaciation, skin lesions (nodules), snoring, nasal discharge, epistaxis, lacrimation, enlarged lymph nodes, anorexia, lower leg edema, pneumonia, low milk yield, and diarrhea. Among these signs, fever, emaciation, lesions on the skin (nodules), and snoring were observed more frequently. Skin nodules were observed over the body, including the neck, chest, abdomen, limbs, and nasal region. Nasal discharge was present in all infected cases (68/68; 100%, 95% CI: 94.7–100). Conversely, absence of nasal discharge was observed in only 49 infected animals (22.6%, 95% CI: 17.5–28.6) compared with 168 uninfected animals (77.4%, 95% CI: 71.4–82.5). A similar pattern was noted for epistaxis, fever, lacrimation, emaciation, enlarged lymph nodes, anorexia, skin lesions, snoring, lower leg edema, and pneumonia, which were recorded only in infected animals, ranging from 27 cases with epistaxis (100%, 95% CI: 87.5–100) to 111 cases with skin lesions (100%, 95% CI: 96.7–100) (Table 2).

**Table 2:** Distribution of clinical signs and symptoms among infected and uninfected animals, showing the number of cases (n), corresponding percentages with 95% confidence intervals (CI), and p-values as determined using the chi-square test.

Status	Infected (*n*)	Infected % (*95% CI*)	Uninfected (*n*)	Uninfected % (*95% CI*)	*p*-value
**Nasal Discharge**					
Absent	49	22.6% (17.5% − 28.6%)	168	77.4% (71.4% − 82.5%)	<0.0001
Present	68	100% (94.7% − 100%)	0	0% (0% − 5.3%)	
**Epistaxis**					
Absent	90	34.9% (29.3% − 40.9%)	168	65.1% (59.1% − 70.7%)	<0.0001
Present	27	100% (87.5% − 100%)	0	0% (0% − 12.5%)	
**Fever**					
Absent	22	11.6% (7.8% − 16.9%)	168	88.4% (83.1% − 92.2%)	<0.0001
Present	95	100% (96.1% − 100%)	0	0% (0% − 3.9%)	
**Lacrimation**					
Absent	51	23.3% (18.2% − 29.3%)	168	76.7% (70.7% − 81.8%)	<0.0001
Present	66	100% (94.5% − 100%)	0	0% (0% − 5.5%)	
**Emaciation**					
Absent	14	7.7% (4.6% − 12.5%)	168	92.3% (87.5% − 95.4%)	<0.0001
Present	103	100% (96.4% − 100%)	0	0% (0% − 3.6%)	
**Enlarged Lymph Nodes**					
Absent	70	29.4% (24% − 35.5%)	168	70.6% (64.5% − 76%)	<0.0001
Present	47	100% (92.4% − 100%)	0	0% (0% − 7.6%)	
**Anorexia**					
Absent	44	20.8% (15.8% − 26.7%)	168	79.2% (73.3% − 84.2%)	<0.0001
Present	73	100% (95% − 100%)	0	0% (0% − 5%)	
**Lesions on Skin (Nodules)**					
Absent	6	3.4% (1.6% − 7.3%)	168	96.6% (92.7% − 98.4%)	<0.0001
Present	111	100% (96.7% − 100%)	0	0% (0% − 3.3%)	
**Snoring**					
Absent	23	12% (8.2% − 17.4%)	168	88% (82.6% − 91.8%)	<0.0001
Present	94	100% (96.1% − 100%)	0	0% (0% − 3.9%)	
**Lower leg edema**					
Absent	58	25.7% (20.4% − 31.7%)	168	74.3% (68.3% − 79.6%)	<0.0001
Present	59	100% (93.9% − 100%)	0	0% (0% − 6.1%)	
**Pneumonia**					
Absent	45	21.1% (16.2% − 27.1%)	168	78.9% (72.9% − 83.8%)	<0.0001
Present	72	100% (94.9% − 100%)	0	0% (0% − 5.1%)	

### 3.3 Correlation analysis of clinical signs and symptoms

The correlations between clinical signs established strong positive relationships among the major symptoms. Skin lesions were strongly related to emaciation (r = 0.87), fever (r = 0.79), and snoring (r = 0.83), indicating a concentration of systemic and respiratory signs with dermatological manifestations. Fever had a positive correlation with various signs, such as snoring (r = 0.71), anorexia (r = 0.61), and pneumonia (r = 0.60), suggesting extensive participation in the pathology of the disease development. Diarrhea had moderate correlations with anorexia (r = 0.65), fever (r = 0.57), and skin lesions (r = 0.70), and thus it could be considered as a secondary sign of systemic infection or stress, as shown in Fig 1. Lower leg edema and enlarged lymph nodes were less associated with other symptoms, but they also suggested a multi-organ effect.

**Fig 1:**
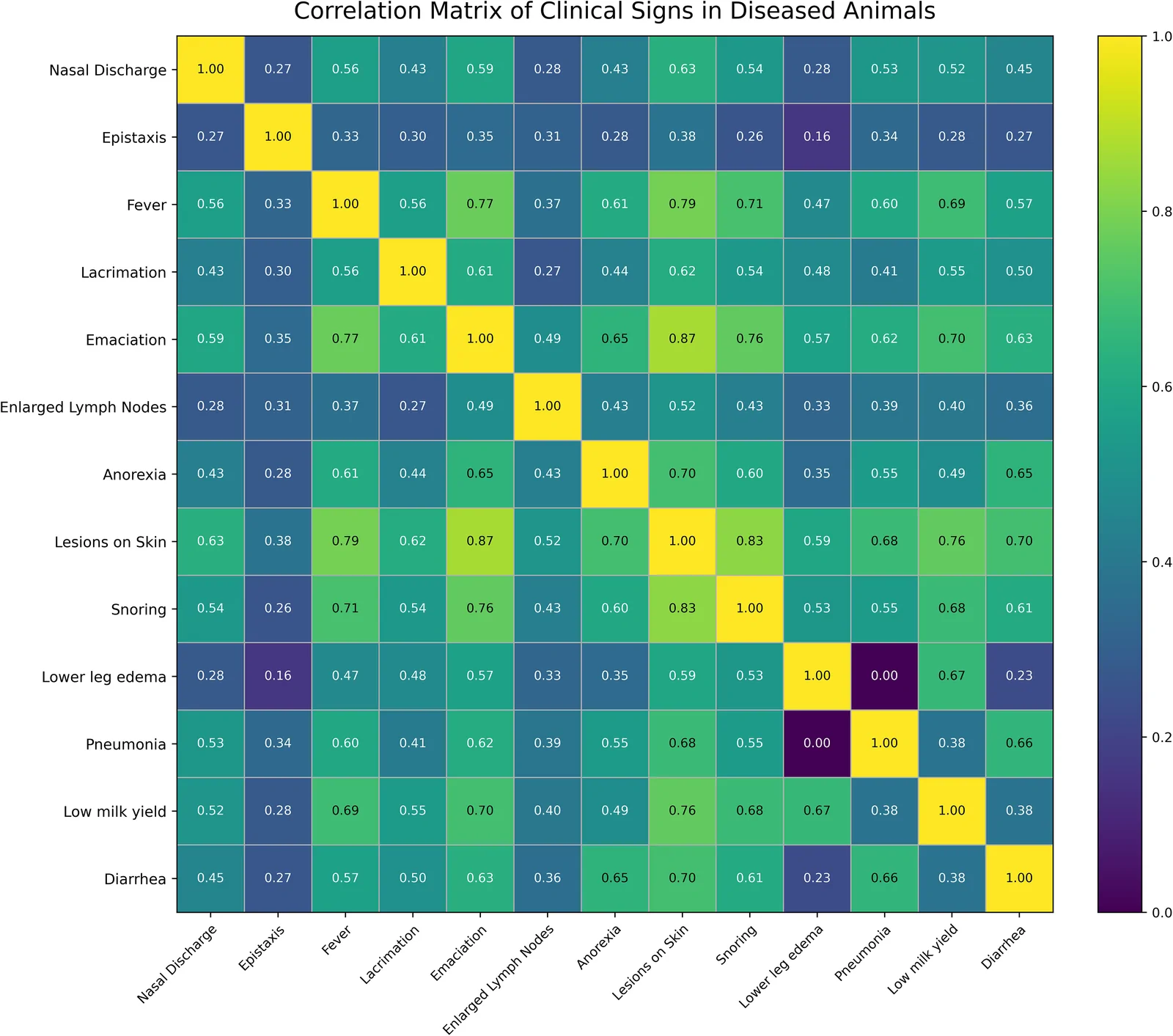
Correlation matrix highlighting the associations’ strength among observed clinical signs in affected animals. Pearson’s correlation coefficients (r) are displayed using a color gradient, where darker colors indicate stronger positive or negative correlations. The scale ranges from −1 to +1, showing a negative to positive association, respectively. The figure was generated using RStudio (version 4.3.1).

### 3.4 Risk factors analysis

The assessment of the risk factors for Lumpy skin disease is based on observational studies. According to the data collected during the study, different risk factors found critical in LSD transmission include vectors (ticks and mosquitoes), shared water sources, no vaccination, mixed animal populations, lack of disinfection, improper manure management, farmer illiteracy, newly purchased animals, behavior based on superstitions, and animals being treated by quacks. Interventions by unqualified practitioners (quacks) were identified as a primary risk factor, as these often delayed professional veterinary care, involved improper lesion management, and compromised biosecurity, thereby facilitating disease transmission. Ticks and mosquitoes were observed on almost all the farms. These can travel to closely located farms or even longer distances via transport trucks used by farmers routinely at farms. These factors can be significant sources of transmission. For vector exposure, all animals with reported presence of vectors were infected (69/69; 100%, 95% CI: 94.7–100), whereas animals not exposed to vectors were largely uninfected (168/216; 77.8%). Age-wise, animals >2 years were exclusively infected (28/28; 100%, 95% CI: 87.9–100). Similarly, those which were kept in mixed populations, belonging to illiterate farmers, newly purchased animals, animals owned by farmers believing in superstitions, and animals treated by quacks all showed 100% infection (ranges of 95% CI: 82.4–100–96–100) (Table 3).

**Table 3:** Comparative distribution of selected risk factors across infected and uninfected animals, presenting counts (n), percentages with 95% confidence intervals (CI), and p-values obtained using the chi-square test for differences in infection status within each category.

Status	Infected (*n*)	Infected % (*95% CI*)	Uninfected (*n*)	Uninfected % (*95% CI*)	*p*-value
Vectors (Ticks, Mosquitoes, etc.)					
Absent	48	22.2% (17.2% − 28.2%)	168	77.8% (71.8% − 82.8%)	<0.0001
Present	69	100% (94.7% − 100%)	0	0% (0% − 5.3%)	
Age					
<2yrs	89	34.6% (29.1% − 40.6%)	168	65.4% (59.4% − 70.9%)	<0.0001
>2yrs	28	100% (87.9% − 100%)	0	0% (0% − 12.1%)	
Water source					
Clean	76	100% (95.2% − 100%)	0	0% (0% − 4.8%)	<0.0001
Unclean	41	19.6% (14.8% − 25.5%)	168	80.4% (74.5% − 85.2%)	
Vaccination					
No	84	33.3% (27.8% − 39.4%)	168	66.7% (60.6% − 72.2%)	<0.0001
Yes	33	100% (89.6% − 100%)	0	0% (0% − 10.4%)	
Mixed population					
No	46	21.5% (16.5% − 27.5%)	168	78.5% (72.5% − 83.5%)	<0.0001
Yes	71	100% (94.9% − 100%)	0	0% (0% − 5.1%)	
Disinfection					
No	99	37.1% (31.5% − 43%)	168	62.9% (57% − 68.5%)	<0.0001
Yes	18	100% (82.4% − 100%)	0	0% (0% − 17.6%)	
Manure management					
No	91	35.1% (29.6% − 41.1%)	168	64.9% (58.9% − 70.4%)	<0.0001
Yes	26	100% (87.1% − 100%)	0	0% (0% − 12.9%)	
Farmer’s literacy					
No	91	35.1% (29.6% − 41.1%)	168	64.9% (58.9% − 70.4%)	<0.0001
Yes	26	100% (87.1% − 100%)	0	0% (0% − 12.9%)	
Newly purchased animals					
No	46	21.5% (16.5% − 27.5%)	168	78.5% (72.5% − 83.5%)	<0.0001
Yes	71	100% (94.9% − 100%)	0	0% (0% − 5.1%)	
The farmer believe in superstitions					
No	28	14.3% (10.1% − 19.9%)	168	85.7% (80.1% − 89.9%)	<0.0001
Yes	89	100% (95.9% − 100%)	0	0% (0% − 4.1%)	
Treatment by Quacks					
Quack	93	100% (96% − 100%)	0	0% (0% − 4%)	<0.0001
Vet	24	12.5% (8.5% − 17.9%)	168	87.5% (82.1% − 91.5%)	

### 3.5 Correlation analysis of Risk factors

Preventive and risk factor correlation analysis also indicated troubling trends in the data, as shown in Fig 2. There was a very strong positive relationship between fictional beliefs and treatment by quacks (r = 0.68), unsafe water sources (r = 0.59), the purchase of new animals (r = 0.56), and a mixed population of households (r 2016 = 0.61). Treatment by quacks was highly correlated with vectors (r = 0.62), fiction believers (r = 0.68), and water sources (r = 0.56), suggesting an association between misinformation, unqualified veterinary care and several identified risk factors, as shown in Fig 3. In contrast, potential effects such as positive practices of disinfection, manure management, and farmer literacy showed lower correlation, indicating a deficiency in awareness of the disease and its control measures.

**Fig 2:**
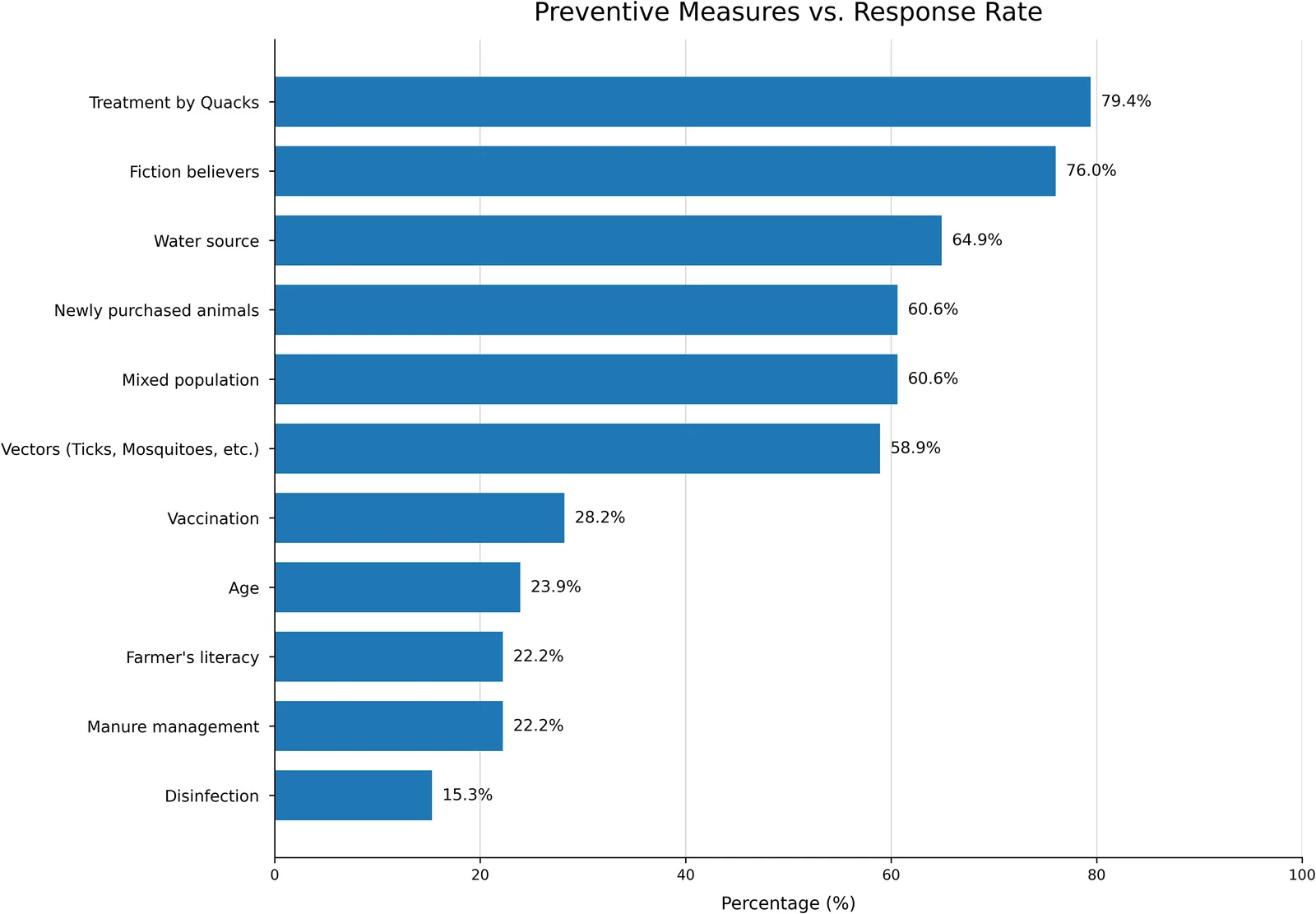
Percentage of responses among farm owners showing preventive measures and key risk factors associated with disease incidence in animals. The data represent responses obtained through structured owner interviews during outbreak investigations.

**Fig 3:**
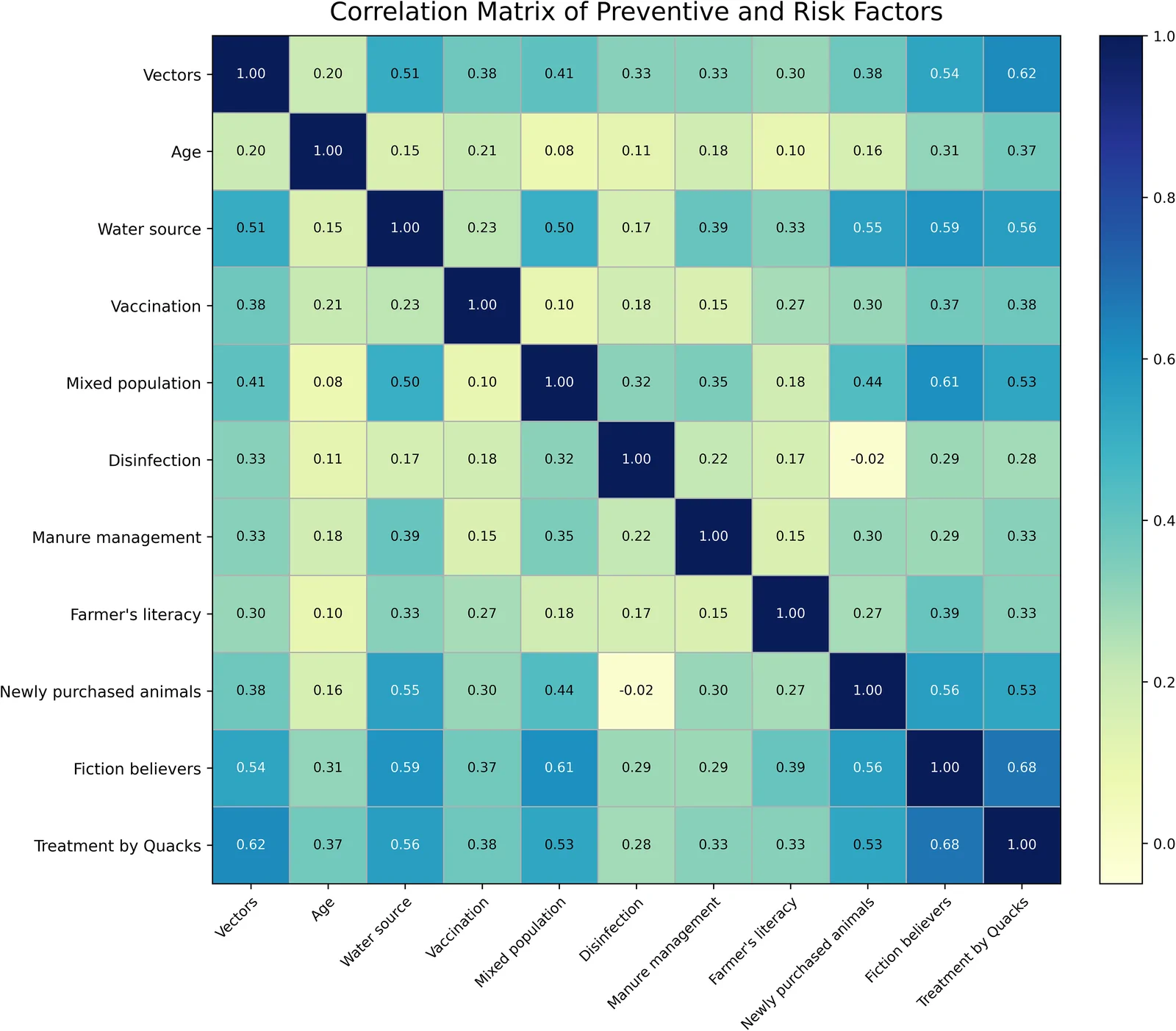
Correlation matrix showing the Pearson correlation coefficients between various selective preventive measures and risk factors observed in diseased animal populations. The color intensity gradient shows the direction and strength of association, with a darker color indicating stronger association. The scale ranges from −1 to +1, showing a negative to positive association, respectively. Visualization was performed using RStudio (version 4.3.1).

### 3.6 Molecular confirmation via the partial RPO30 gene

PCR amplification of the partial RPO30 gene, indicated by a band of 554 base pairs, confirmed the presence of the lumpy skin disease virus (LSDV) DNA in all samples (Fig 4). Among the clinically suspected cases included in this study, more than 90% were PCR positive for, which revealed the extensive spread of the virus in the regions of occurrence. Skin scab samples showed the highest positivity rates (85.9%), whereas blood samples showed a lower but significant positivity rate (69.2%).

**Fig 4:**
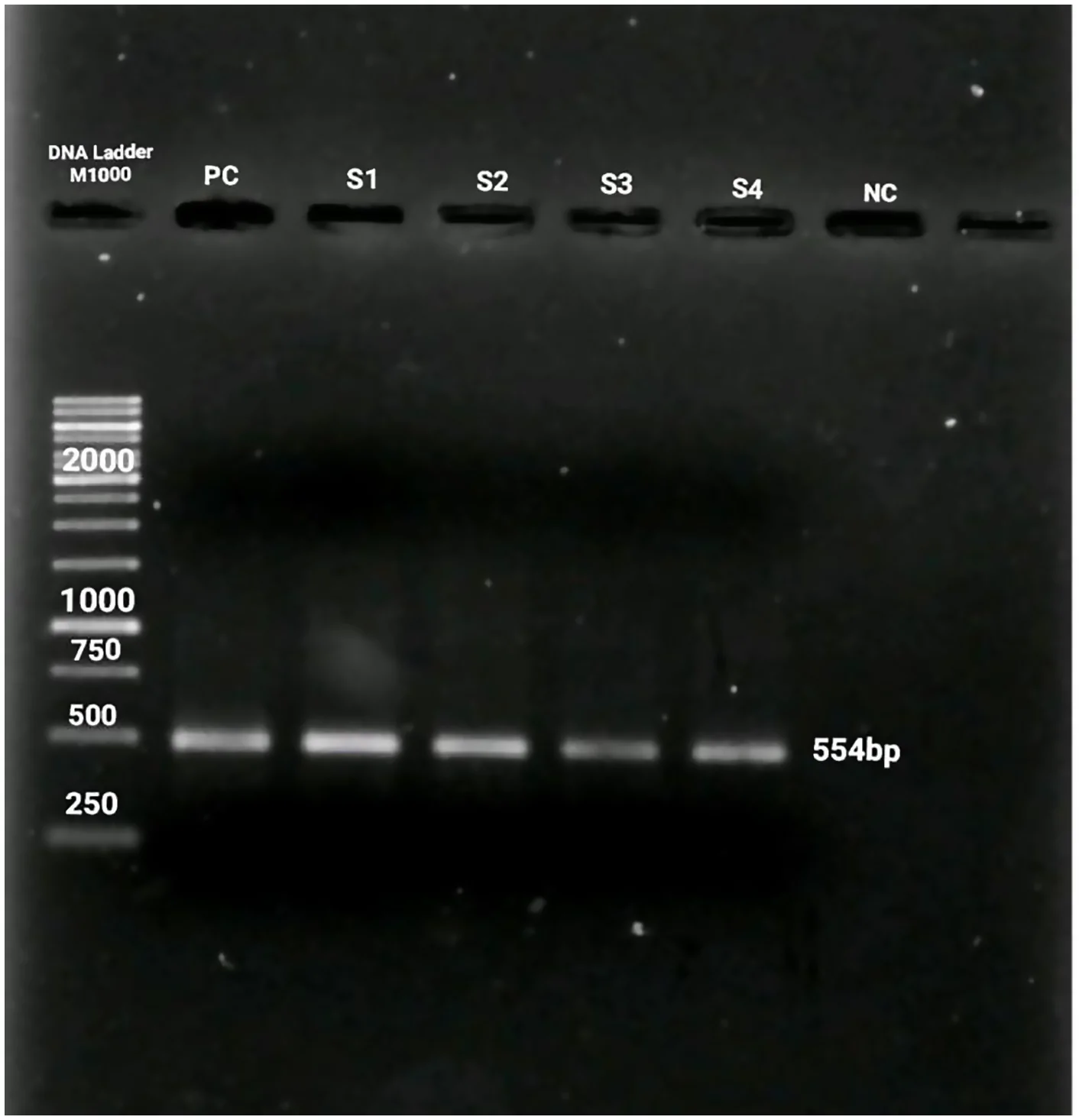
Gel electrophoresis showing a 554 bp amplicon of the *RPO30* gene. DNA ladder: 1 kb, PC: positive control, Lane 1-4: LSDV positive samples, NC: negative control.

The seven selected representative samples that had undergone PCR-positive tests in geographically different locations were sequenced to measure genetic diversity and evolutionary relationships.

### 3.7 Phylogenetic analysis of LSDV

The generated partial 554-bp fragment of the RPO30 gene sequences were submitted to GenBank and are now available in the NCBI database, having accession numbers OR745274–OR745280 (Table 4). Reference sequences from neighboring countries were included to assess the phylogenetic relationship of Pakistani LSDV isolates and to explore the potential transboundary transmission pattern associated with the outbreak.

**Table 4:** Comparative RPO30 gene accession numbers of LSDV isolates from Pakistan (2022 Cholistan outbreak) with regional reference strains from China (2019-2023), Bangladesh (2019-2022), and India (2019-2022).

Area	Year	Host specie	Accession No
Pakistan	2022 2022 2022 2022 2022 2022 2022 2022 2022 2022 2022	LSDV	OR745274 23/IUB/PK OR745275 25/IUB/PK OR745276 L4/IUB/PK OR745277 L2/IUB/PK OR745278 P7/IUB/PK OR745279 MB/IUB/PK OR745280 M1/IUB/PK OP381107 MG/PAK/2022 OR393303 RSL4/NIAB/PAK OR393304 RP7/NIAB/PAK OR393305 RMB1/NIAB/PAK
China	2019 2019 2019 2020 2020 2021 2023	LSDV	MN518933 China/XJ/2019 MN598007 China/Xinjiang/2019 MN864146 China/XJ/2019 OL977076 China/NMG/2020 MW251476 China/Guangdong/2020 ON691457 China/SiC03/2021 OL602179 China/Xinjiang/2023
Bangladesh	2019 2022	LSDV	MT448696 BAN Patna 2019 OM802146 NIB/Dinajpur14/2022
India	2019 2019 2019 2020 2020 2022 2022 2022 2022 2023 2023	LSDV	MW452636 IND/WB0S10-LT/2019 MW452638 IND/WB0GS2-LT/2019 MT074114 IND/ODI/8PK/2019 OM362844 Chiplum-14/2020 OM362835 malligere-MD/2020 OQ943603 India/Haryana/2022 OP903458 NIVEDI-LSDV-Haveri 1 OQ030223 TN/CUL/TVLR/2022 OQ030222 India TN/CUL/Kris/2022 OR678467 India/Gujrat/2023 OR689755 India/Gujrat/2023

The phylogenetic tree (Fig 5) showed that the LSDV isolates in the current study were closely clustered with LSDV strains reported in Asia (India, Bangladesh, and China), implying a close evolutionary relationship. The observed genetic similarity suggests that Pakistani isolates are closely related to other regional LSDV strains. Detailed sequence metadata are provided in Supplementary S3 File.

**Fig 5:**
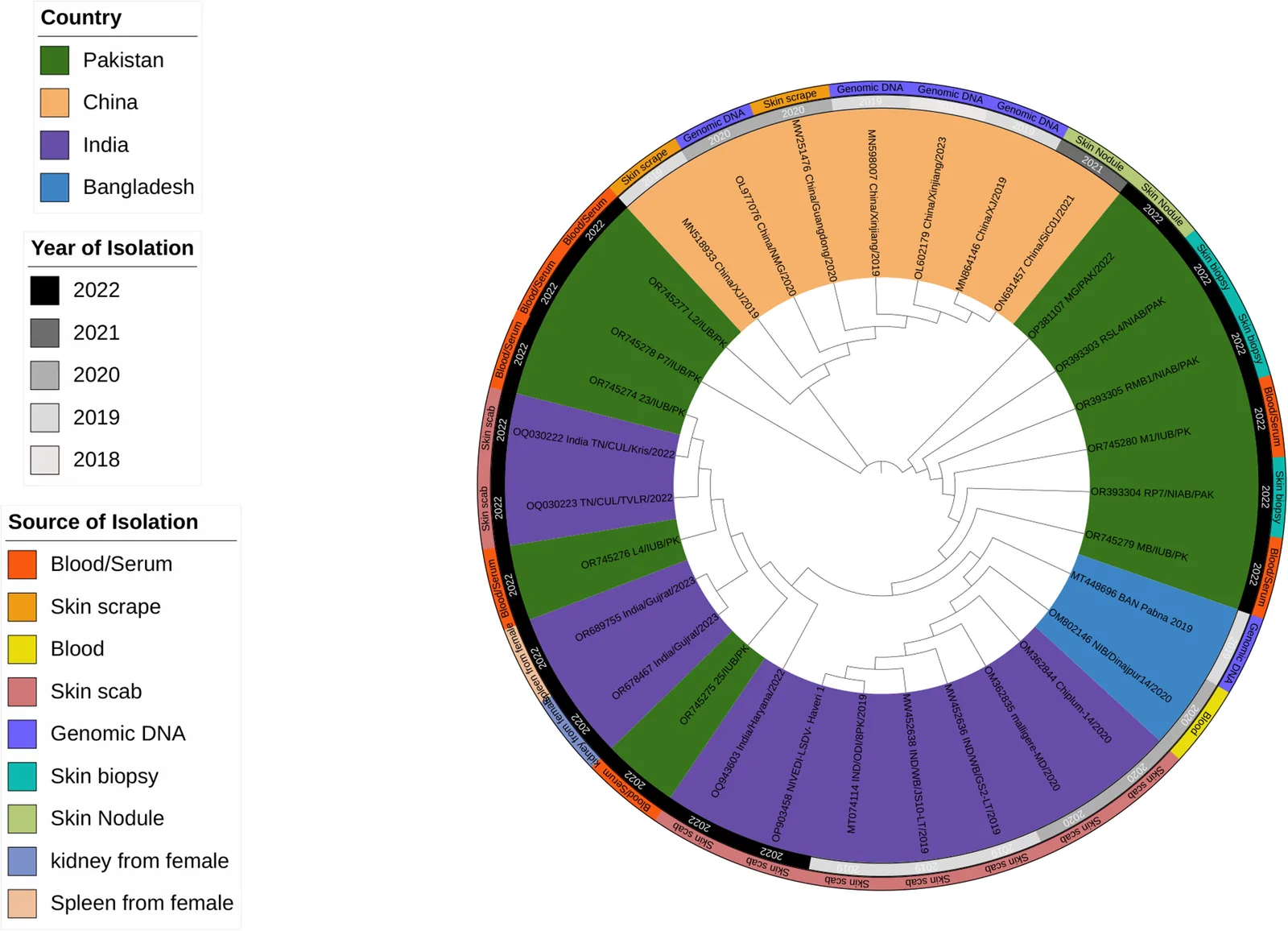
Phylogenetic tree showing the relationship between LSDV *RPO30 gene* sequences from Pakistan, with other Capripoxvirus RPO30 gene sequences from GenBank. Numbers indicate the values calculated at 1000 bootstrap replicates.

## 4 Discussion

The present study provides an integrated epidemiology, molecular characterization, and risk factor analysis of lumpy skin disease in cattle from selected regions of Pakistan. The field based investigation combines with molecular and risk factor analysis provides a comprehensive understanding disease occurrence and management related problems. Several management- and behavior-related factors showed significant associations with lumpy skin disease occurrence. In particular, treatment by unqualified practitioners and limited implementation of biosecurity measures were strongly associated with infection status. One plausible explanation is that delayed veterinary consultation and inappropriate handling of infected animals may prolong viral persistence at the farm level, increasing opportunities for transmission to susceptible cattle. These findings suggest that sociocultural and management practices may be associated with outbreak occurrence and demand further investigation.

Overall, the findings of this study should be interpreted in the context of a field based outbreak investigation, where disease dynamics showed the combined influence of host susceptibility, management practices, and environmental exposure. These factors likely interact to shape transmission patterns at the herd level in livestock production system.

The observed clinical manifestations were consistent with the published descriptions of clinical manifestations in other endemic areas: skin nodules were in 94.8% of cases, which is consistent with the existing data of skin nodules as the pathogenic sign of the disease in Bangladesh and Iraq (where cutaneous lesions are the pathogenic sign) [38], and the high prevalence of fever (83.7%), emaciation (88%), enlarged lymph nodes (40.1%), and diarrhea (60.6%) confirm this pattern and indicate that these patterns of susceptibility were also consistent with the earlier reports, young animals, dairy cattle, and cross breeds exhibited preferential infection, which is similar to those found in Bangladesh and suggesting that these factors may be consistently associated with disease occurrence across different geographic regions [38–41]. The frequency of skin lesions as the primary clinical sign reinforce their diagnostic relevance under field conditions, particularly in resource-limited settings where laboratory confirmation may be delayed, supporting early case identification and outbreak response.

Molecular characterization based on partial RPO30 gene sequencing revealed close genetic relatedness among circulating strains and clustering with previously reported regional isolates. Although the level of genetic variation observed was limited, the selected target gene provides sufficient discriminatory power for lineage assignment within the Capripoxvirus genus. These findings are consistent with regional transmission patterns and are consistent with epidemiological observations recorded during the study, while avoiding over-interpretation beyond the resolution of partial gene sequencing. The results of our > 90% PCR positivity and the capacity to distinguish LSDV from other related poxviruses are supported by the results of Uganda and other countries, making this target worthwhile for international surveillance [42]. Likewise, in line with this, our molecular characterization of clustering Pakistani isolates closely with Asian strains in India, Bangladesh, and China is in good agreement with prior studies that are consistent with previous reports describing genetic similarity among LSDV strains circulating in several Asian countries [43–45]. From an epidemiological perspective, the higher positivity in skin scab samples compared to blood highlighted the importance of sample selection for accurate laboratory diagnosis.

The large decrease in milk production (75.2) and moderate to high prevalence of pneumonia (61.5) are similar to the economic impacts of the endemic regions and confirm that production loss is a major burden [46]. Most importantly, our statistical demonstration that the illiteracy of the farmers (65%), non-scientific disease beliefs (76.0%), and quack treatment (79.4%) are factors significantly associated with LSDV transmission (p < 0.0001) is a great deviation because most of the previous research has centered on vector ecology, biosecurity failures, and animal trade as the key determinants. Although prior studies in Bangladesh, Iraq, and Ethiopia have reported clinical and veterinary determinants of LSD, sociocultural determinants have been neglected, although our null-hypothesis findings of strong relationships between fictional beliefs and quack treatment (r = 0.68), unsafe water sources (r = 0.59), and animal introduction (r = 0.56) indicate that they are not confounding variables but potentially important epidemiological factors in resource-constrained environments [38,40]. This result is contrary to the underlying notion of the majority of earlier research that control failures are only caused by technical constraints and not behavioral ones (12). Thus, there can be no misinformation to overturn more traditional interventions, even in the presence of such services. Equally inconsistent is our finding that was only statistically significant when combined with biosecurity failures (p > 0.05, 58.9%), in contrast to systematic reviews highlighting the importance of stable flies (Stomoxys calcitrans) and biting midges as the primary agents of transmission [47].

Although we found LSDV on farms, which is in agreement with what is already known, we found that vector-related variables were not significantly associated with infection status in the present study unless accompanied by biosecurity failures, which is inconsistent with the literature indicative of the effectiveness of vector control alone. The fact that we did not find significant variation between vaccination status (p > 0.05, 28.2% prevalence) and disease occurrence is not consistent with a highly vaccinated area where campaigns changed the epidemiology [46], even though this interpretation must reflect low vaccine availability and not low vaccine efficacy, which is contrary to the assumptions that vaccination could be sustained in the context of weak infrastructure, where it becomes secondary to underlying biosecurity failures [48].

Moreover, our observation that pneumonia (61.5%) and respiratory manifestations such as snoring (83.7%) were remarkably high is not consistent with the majority of past reports, where respiratory manifestations were not so actively displayed. This could be an indicator of virulence strain-specificity or environmental factors that predispose patients to respiratory complications and are worthy of study [49]. On the other hand, our result of 64.9% of diseased animals getting unsafe water and poor manure management was high, which is consistent with the risk factor analysis in Bangladesh and Uganda [46,50]. However, unlike earlier studies, which treated them as minor contributors to the spread, our findings identified these among the factors most strongly associated with infection status. The observed association between management practices and disease occurrence suggest that LSD transmission in study is strongly shaped by farm-level behavior and structural conditions. These findings indicates that control measures should extend beyond biological interventions alone and incorporate improvements in farming practices, veterinary access and routine biosecurity implementations.

The relatively wide confidence intervals recorded for some risk factors estimates likely reflects the limited sample size with certain subgroups and the inherent heterogeneity of field-based outbreak data. This reduced precision should be considered when interpreting the strength of association reported in the present study. However, the directions of observed effects remained consistent across variables, supporting the overall robustness of the findings.

Our novel correlation analysis, which has shown interconnections between fictional beliefs, quack treatment, unsafe water sources, and the introduction of animals to the environment, is an analytical method that has not been highlighted in the literature on LSD, where previous studies have defined them as individual risk factors [46]. However, our quantitative results of interdependence between these areas imply that intervention in a single domain can propagate to others [51], which contradicts the implied compartmentalization of other analytical methods. These findings contribute additional evidence regarding the relative importance of different risk factors on LSD: several studies find that vectors are important but our data demonstrate that vectors are not significantly predictive at the expense of other factors; African and Middle Eastern research finds lower morbidity-mortality despite the presence of vectors, but lacks vaccination, indicating that previous studies may have underestimated the potential of the trait of high morbidity-mortality in areas with low biosecurity and low knowledge, and therefore the importance of vectors and vaccination are critical predictors in well-managed farm environments; so well-managed areas of developed Our findings imply, therefore, that in the context of Pakistan and other low-resource areas, the control of LSD should be context-calibrated: as much as the use of vectors and vaccination was used in the past as an appropriate intervention in well-resourced areas with sufficient levels of farmer literacy [47], our results suggest that in Pakistan and other low-resource settings, the problem of farm misinformation has to be managed and biosecurity promoted before vaccination can reach full protective potentials. Importantly, the findings highlight that effective control of LSD in endemic, low resource setting requires an integrated approach that combines vaccination, vector management and strengthening of farmer knowledge and biosecurity practices.

This study has certain limitations that should be acknowledged. Risk factor data were collected retrospectively through field-based interviews, which may be subject to recall bias. However, this was mitigated by conducting interviews shortly after outbreak detection using a structured questionnaire and by cross-verifying responses with clinical observations wherever possible. Additionally, some epidemiological variables, including viral exposure and seasonal factors, were not consistently available across all farms and therefore could not be comprehensively analyzed. Molecular characterization was based on partial gene sequencing rather than whole genome sequencing, which may limit resolution of genetic diversity. Nevertheless, the selected genetic target provides reliable lineage discrimination and supports the epidemiological interpretations presented.

## 5 Conclusion

The current study examined the LSDV infection outbreak in the Cholistan region of Pakistan, revealing the overall clinical signs and symptoms of the disease and its risk factors. Additionally, partial RPO30 gene sequencing demonstrated close genetic similarity between Pakistani isolates and previous reported regional LSDV. The information produced by this study will help Pakistani field veterinarians and decision-makers in the field of animal health to take the necessary actions to stop future outbreaks or recurrence of the disease.

## Supporting information

S1 File Consent form. The consent form used to obtain verbal consent from livestock owners participating in the study.
(DOC)

S2 File LSDV questionnaire. Questionnaire used to collect epidemiological data from livestock owners during the study.
(DOCX)

S3 File Lumpy skin disease sequnces for phylogeny. (DOCX)
