# Supplementary material for: Retrospective analysis of genetic diversity, clinical manifestations and risk factors, during outbreak of lumpy skin disease virus
Source: PLoS One. 2026 Oct 16;21(10):e0358559. doi: 10.1371/journal.pone.0358559 (PMC13674707; doi:10.1371/journal.pone.0358559)
Supplement: Supplementary file 1 [file pone.0358559.s002.docx]

**QUESTIONNAIRE**

**Study title:**

**Retrospective analysis of genetic diversity, clinical manifestations and risk factors, during outbreak of lumpy skin disease virus**

**BIOTIC FACTORS (Living)**

**Sample type:** Blood

**Age:** < 1 year, >1 to ≤3 year or >3 to 6 year___________

**Gender:** Male, female_____________________________

**Breed:** Local, cross or exotic________________________

**Insect vectors:** Present and absent ___________________

**ABIOTIC FACTORS (Non-living)**

**Area:** _____________________

**Grazing system:** Separate or communal_______________

**Health status:** Good, moderate or poor _______________

**Vectors**: (Ticks, Mosquitoes, etc.) ___________________

**Water Source:** Clean, unclean ___________________

**Mixed Population:** Yes, No ___________________

**Disinfection:** Yes, No ___________________

**Manure management:** Yes, No ___________________

**Farmer's literacy:** Yes, No ___________________

**Newly purchased animals:** Yes, No ___________________

**Farmers believed in superstitions:** Yes, No ___________________

**Treatment by quacks:** Quack, Vet ___________________
