# Supplementary material for: Retrospective analysis of genetic diversity, clinical manifestations and risk factors, during outbreak of lumpy skin disease virus
Source: PLoS One. 2026 Oct 16;21(10):e0358559. doi: 10.1371/journal.pone.0358559 (PMC13674707; doi:10.1371/journal.pone.0358559)
Supplement: Supplementary file 2 [file pone.0358559.s003.docx]

>OR745280.1 Lumpy skin disease virus LSDV/M1/IUB/PK RNA polymerase subunit 30 kDa (RPO30) gene, partial cds

ATGGATGATGATAATACTAATTCATATAGTGATAATACTACCCCCACATATCAAGACATAGAAGATATAATTTAAAATATGTAAAAGAAAAATCAAAGGTAAAAGAAATATTAAAATGGGCAACAGACAAAGCTTCCAAGTTTTATATAGAAATATTATTAATACAAAGTCAAATATAGAAGAAACAAAATTTGAACCAAGAAACAACATAGGTATTGAATATCAAAAGATTCAAAAAACAAATTATCGTATAGAAACAAGCCTTTAATAGAGACAAATAAAGATTATTCTGACATTGTGATCTTATACGTACGACAAATGGAACAGAGAAAGAAATTTTAAGATATATACTTTTTGGAATAAAATGTGTCAAAAAAATGTAGAATTCAATATAGACAATATTAGAGATATAAATCACGAAGAATATTTTAATGTTTTAGATAAAAAGTATAACCTCCCATGCCCTGAGTGTAAAAGTAAAAACACTATTCCCGTCATGATACAAACAAGAGCAGCAGATGAACCACCATTAGT

>OR393305.1 Lumpy skin disease virus isolate LSDV/RMB1/NIAB/PAK RNA polymerase subunit 30 (RPO30) gene, partial cds

ATGGATGATGATAATACTAATTCATATAGTGATAATACTACCCCCACATATCAAGACATAGAAGATATAATTTATAAATATGTAAAAGAAAAATCAAAGGTAAAAGAAATATTAAAATGGGCAACAGACAAAGCTTCCAAGTTTTATATAAGAAATATTATTAATACAAAGTCAAATATAGAAGAAACAAAATTTGAACCAAGAAACAACATAGGTATTGAATACTCAAAAGATTCAAAAAACAAATTATCGTATAGAAACAAGCCTTTAATAGAGACAAATAAAGATTATTCTGACATATGTGATCTTATACGTACGACAAATGGAACAGAGAAAGAAATTTTAAGATATATACTTTTTGGAATAAAATGTGTTCAAAAAAATGTAGAATTCAATATAGACAATATTAGAGATATAAATCACGAAGAATATTTTAATGTTTTAGATAAAAAGTATAACCTCCCATGCCCTGAGTGTAAAAGTAAAAACACTATTCCCGTCATGATACAAACAAGAGCAGCAG

>OR393303.1 Lumpy skin disease virus isolate LSDV/RSL4/NIAB/PAK RNA polymerase subunit 30 (RPO30) gene, partial cds

ATGGATGATGATAATACTAATTCATATAGTGATAATACTACCCCCACATATCAAGACATAGAAGATATAATTTATAAATATGTAAAAGAAAAATCAAAGGTAAAAGAAATATTAAAATGGGCAACAGACAAAGCTTCCAAGTTTTATATAAGAAATATTATTAATACAAAGTCAAATATAGAAGAAACAAAATTTGAACCAAGAAACAACATAGGTATTGAATACTCAAAAGATTCAAAAAACAAATTATCGTATAGAAACAAGCCTTTAATAGAGACAAATAAAGATTATTCTGACATATGTGATCTTATACGTACGACAAATGGAACAGAGAAAGAAATTTTAAGATATATACTTTTTGGAATAAAATTGTTCAAAAAAATGTAGAATTCAATATAGACAATATTAGAGATATAAATCACGAAGAATATTTTAATGTTTTAGATAAAAAGTATAACCTCCCATGCCCTGAGTGTAAAAGTAAAAACA

C

>OP381107.1 Lumpy skin disease virus isolate LSDV/MG/PAK/2022 RNA polymerase subunit 30 kDa (RPO30) gene, partial cds

ATGGATGATGATAATACTAATTCATATAGTGATAATACTACCCCCACATATCAAGACATAGAAGATATAATTTATAAATATGTAAAAGAAAAATCAAAGGTAAAAGAAATATTAAAATGGGCAACAGACAAAGCTTCCAAGTTTTATATAAGAAATATTATTATTACAAAGTCAAATATAGAAGAAACAAAATTTGAACCAAGAAACAACATAGGTATTGAATACTCAAAAGATTCAAAAAACAAATTATCGTATAGAAACAAGCCTTTAATAGAGACAAATAAAGATTATTCT

>OR745274.1 Lumpy skin disease virus LSDV/23/IUB/PK RNA polymerase subunit 30 kDa (RPO30) gene, partial cds

ATGGATGATGATAATACTAATTCATATAGTGATAATACTAACCCCACATATCAAGACATAGAAGATATAATTTATAAATATGTAAAAGAAAAATCAAAGGTAAAAGAAATATTAAAATGGGCAACAGACAAAGCTTCCAAGTTTTATATAAGAAATATTATTAATACAAAGTCAAATATAGAAGAAACAAAATTTGAACCAAGAAACAACATAGGTATTGAATACTCAAAAGATTCAAAAAACAAATTATCGTATAGAAACAAGCCTTTAATAGAGACAAATAAAGATTATTCTGACATATGTGATCTTATACGTACGACAAATGGAACAGAGAAAGAAATTTTAAGATATATACTTTTTGGAATAAAATGTGTTCAAAAAAATGTAGAATTCAATATAGACAATATTAGAGATATAAATCACGAAGAATATTTTAA

>OR745275.1 Lumpy skin disease virus LSDV/25/IUB/PK RNA polymerase subunit 30 kDa (RPO30) gene, partial cds

ATGGATGATGATAATACTAATTCATATAGTGATAATACTAACCCCACATATCAAGACATAGAAGATATAATTTATAAATATGTAAAAGAAAAATCAAAGGTAAAAGAAATATTAAAATGGGCAACAGACAAAGCTTCCAAGTTTTATATAAGAAATATTATTAATACAAAGTCAAATATAGAAGAAACAAAATTTGAACCAAGAAACAACATAGGTATTGAATACTCAAAAGATTCAAAAAACAAATTATCGTATAGAAACAAGCCTTTAATAGAGACAAATAAAGATTATTCTGACATATGTGATCTTATACGTACGACAAATGGAACAGAGAAAGAAATTTTAAGATATATACTTTTTGGAATAAAATGTGTTCAAAAAAATGTAGAATTCAATATAGACAATATTAGAGATATAAATCACGAAGAATATTTTAATGTTTTAGATAAAAAGTATAACCTCCCATGCCCTGAGTGTAAAAGTAAAAACACTATTCCCGTCATGATACAAACAAGAGCAGCAGATGAACCACCATTAGTTATGCATTCTT

>OR745276.1 Lumpy skin disease virus LSDV/L4/IUB/PK RNA polymerase subunit 30 kDa (RPO30) gene, partial cds

ATGGATGATGATAATACTAATTCATATAGTGATAATACTAACCCCACATATCAAGACATAGAAGATATAATTTATAAATATGTAAAAGAAAAATCAAAGGTAAAAGAAATATTAAAATGGGCAACAGACAAAGCTTCCAAGTTTTATATAAGAAATATTATTAATACAAAGTCAAATATAGAAGAAACAAAATTTGAACCAAGAAACAACATAGGTATTGAATACTCAAAAGATTCAAAAAACAAATTATCGTATAGAAACAAGCCTTTAATAGAGACAAATAAAGATTATTCTGACATATGTGATCTTATACGTACGACAAATGGAACAGAGAAAGAAATTTTAAGATATATACTTTTTGGAATAAAATGTGTTCAAAAAAATGTAGAATTCAATATAGACAATATTAGAGATATAAATCACGAAGAATATTTTAATGTTTTAGATAAAAAGTATAACCTCCCATGCCCTGAGTGTAAAAGTAAAAACACTATTCCCGTCATGATACAAACAAGAGCAGCAGATGAACCACCATTAGTTATGCATTCTT

>OR745278.1 Lumpy skin disease virus LSDV/P7/IUB/PK RNA polymerase subunit 30 kDa (RPO30) gene, partial cds

ATGAAAAAAAAAAATTTTTTAGTTTTAAATACAACAAAAATAAAAATGTATTCTTGTGATGAAGTAGATTCGTATGAACTTGTGAAAAAAATGGTAAATAATCTATCAGAAAGTGAGTTTATAACAGCAATAGAAATATCTAGAAAACTAAATATTGAAAAATCAAATGTAAACAAACAGCTGTATAAACTACATAATGACGGTTTTATTTTTATGATAAGATCGAATCCACCTAAATGGTTTAAAAAAAATGGCATCGATAACGATGACAATGAGAATAACGATACGAAAAAACTAAATAAATCATTTTCTGATACAATTCCGTACTATAAAATTGTTTTATGGAAAGAAAAAAATCCTTGTTCTGCGATAAACGAAT

>OR745277.1 Lumpy skin disease virus LSDV/L2/IUB/PK RNA polymerase subunit 30 kDa (RPO30) gene, partial cds

ATGGATGATGATAATACTAATTCATATAGTGATAATACTACCCCCACATATCAAGACATAGAAGATATAATTTATAAATATGTAAAAGAAAAATCAAAGGTAAAAGAAATATTAAAATGGGCAACAGACAAAGCTTCCAAGTTTTATATAAGAAATATTATTAATACAAAGTCAAATATAGAAGAAACAAAATTTGAACCAAGAAACAACATAGGTATTGAATACTCAAAAGATTCAAAAAATAAATTATCGTATAGAAACAAGCCTTTAATAGAGACAAATAAAGATTATTCTGACATATGTGATCTTATACGTACGACAAATGGAACAGAGAAAGAAATTTTAAGATATATACTTTTTGGAATAAAATGTGTTCAAAAAAATGTAGAATTCAATATAGACAATATTAGAGATATAAACCACGAAGAATATTTTAATGTTTTAGATAAAAAGTATAACCTCCCATGCCCTGAGTGCAAAAGTAAAAACACTATTCCCGTCATGATACAAACAAGAGCAGCAGATGAACC

>OR745279.1 Lumpy skin disease virus LSDV/MB/IUB/PK RNA polymerase subunit 30 kDa (RPO30) gene, partial cds

ATGGATGATGATAATACTAATTCATATAGTGATAATACTACCCCCACATATCAAGACATAGAAGATATAATTTATAAATATGTAAAAGAAAAATCAAAGGTAAAAGAAATATTAAAATGGGCAACAGACAAAGCTTCCAAGTTTTATATAAGAAATATTATTAATACAAAGTCAAATATAGAAGAAACAAAATTTGAACCAAGAAACAACATAGGTATTGAATACTCAAAAGATTCAAAAAACAAATTATCGTATAGAAACAAGCCTTTAATAGAGACAAATAAAGATTATTCTGACATATGTGATCTTATACGTACGACAAATGGAACAGAGAAAGAAATTTTAAGATATATACTTTTTGGAATAAAATGTGTTCAAAAAAATGTAGAATTCAATATAGACAATATTAGAGATATAAATCACGAAGAATATTTTAATGTTTTAGATAAAAAGTATAACCTCCCATGCCCTGAGTGTAAAAGTAAAAACACTATTCCCGTCATGATACAAACAAGAGCAGCAGATGAACCACCATTAGTTATGCATTCTTGTAGAG

>OR393304.1 Lumpy skin disease virus isolate LSDV/RP7/NIAB/PAK RNA polymerase subunit 30 (RPO30) gene, partial cds

ATGGATGATGATAATACTAATTCATATAGTGATAATACTACCCCCACATATCAAGACATAGAAGATATAATTTATAAATATGTAAAAGAAAAATCAAAGGTAAAAGAAATATTAAAATGGGCAACAGACAAAGCTTCCAAGTTTTATATAAGAAATATTATTAATACAAAGTCAAATATAGAAGAAACAAAATTTGAACCAAGAAACAACATAGGTATTGAATACTCAAAAGATTCAAAAAACAAATTATCGTATAGAAACAAGCCTTTAATAGAGACAAATAAAGATTATTCTGACATATGTGATCTTATACGTACGACAAATGGAACAGAGAAAGAAATTTTAAGATATATACTTTTTGGAATAAAATGTGTTCAAAAAAATGTAGAATTCAATATAGACAATATTAGAGATATAAATCACGAAGAATATTTTAATGTTTTAGATAAAAAGTATAACCTCCCATGCCCTGAGTGTAAAAGTAAAAACACTATTCCCGTCATGATACAAACAAGAGCAGCAGATGAACCACCATTAGTTAT

>MN518933.1 Lumpy skin disease virus strain China/XJ/2019 DNA-dependent RNA polymerase subunit (RPO30) gene, complete cds

ATGGATGATGATAATACTAATTCATATAGTGATAATACTACCCCCACATATCAAGACATAGAAGATATAATTTATAAATATGTAAAAGAAAAATCAAAGGTAAAAGAAATATTAAAATGGGCAACAGACAAAGCTTCCAAGTTTTATATAAGAAATATTATTAATACAAAGTCAAATATAGAAGAAACAAAATTTGAACCAAGAAACAACATAGGTATTGAATACTCAAAAGATTCAAAAAATAAATTATCGTATAGAAACAAGCCTTTAATAGAGACAAATAAAGATTATTCTGACATATGTGATCTTATACGTACGACAAATGGAACAGAGAAAGAAATTTTAAGATATATACTTTTTGGAATAAAATGTGTTCAAAAAAATGTAGAATTCAATATAGACAATATTAGAGATATAAACCACGAAGAATATTTTAATGTTTTAGATAAAAAGTATAACCTCCCATGCCCTGAGTGCAAAAGTAAAAACACTATTCCCGTCATGATACAAACAAGAGCAGCAGATGAACCACCATTAGTTATGCATTCTTGTAGAGACTGCAAGAAAAATTTTAAACCTCCAAAGTTTAGAGCTGTAGAAAAATAA

>MN598007.1 Lumpy skin disease virus isolate LSDV/Xinjiang/2019 RNA polymerase subunit 30 kDa (RPO30) gene, complete cds

ATGGATGATGATAATACTAATTCATATAGTGATAATACTACCCCCACATATCAAGACATAGAAGATATAATTTATAAATATGTAAAAGAAAAATCAAAGGTAAAAGAAATATTAAAATGGGCAACAGACAAAGCTTCCAAGTTTTATATAAGAAATATTATTAATACAAAGTCAAATATAGAAGAAACAAAATTTGAACCAAGAAACAACATAGGTATTGAATACTCAAAAGATTCAAAAAATAAATTATCGTATAGAAACAAGCCTTTAATAGAGACAAATAAAGATTATTCTGACATATGTGATCTTATACGTACGACAAATGGAACAGAGAAAGAAATTTTAAGATATATACTTTTTGGAATAAAATGTGTTCAAAAAAATGTAGAATTCAATATAGACAATATTAGAGATATAAACCACGAAGAATATTTTAATGTTTTAGATAAAAAGTATAACCTCCCATGCCCTGAGTGCAAAAGTAAAAACACTATTCCCGTCATGATACAAACAAGAGCAGCAGATGAACCACCATTAGTTATGCATTCTTGTAGAGACTGCAAGAAAAATTTTAAACCTCCAAAGTTTAGAGCTGTAGAAAAATAA

>MN864146.1 Lumpy skin disease virus isolate China/XJ/flies/2019 RNA polymerase subunit (lw036) gene, complete cds

CACCAACCATAGAATAGTATCGAGACATGCTATTTCTAACTTTTAAAAAACATTCATCTAAATCATATTTTTTTTCGTTATAAAACTGTTTAAATAACCTACATGCATAAACAGAAGCTTTTTCAAAGTGATTAGGTGGTGTAATATTTTCCATTTTTGCCATTAATGGAAAAATCTAAATAAAGAACTAAAAATTCATTTTTGTAAATATTGAAATTAAACAACTTCAGTACAAATATGGATGATGATAATACTAATTCATATAGTGATAATACTACCCCCACATATCAAGACATAGAAGATATAATTTATAAATATGTAAAAGAAAAATCAAAGGTAAAAGAAATATTAAAATGGGCAACAGACAAAGCTTCCAAGTTTTATATAAGAAATATTATTAATACAAAGTCAAATATAGAAGAAACAAAATTTGAACCAAGAAACAACATAGGTATTGAATACTCAAAAGATTCAAAAAATAAATTATCGTATAGAAACAAGCCTTTAATAGAGACAAATAAAGATTATTCTGACATATGTGATCTTATACGTACGACAAATGGAACAGAGAAAGAAATTTTAAGATATATACTTTTTGGAATAAAATGTGTTCAAAAAAATGTAGAATTCAATATAGACAATATTAGAGATATAAACCACGAAGAATATTTTAATGTTTTAGATAAAAAGTATAACCTCCCATGCCCTGAGTGCAAAAGTAAAAACACTATTCCCGTCATGATACAAACAAGAGCAGCAGATGAACCACCATTAGTTATGCATTCTTGTAGAGACTGCAAGAAAAATTTTAAACCTCCAAAGTTTAGAGCTGTAGAAAAATAACATAAACTATAAAAATGAAAAAAAAAAATTTTTTAGTTTTAAATACAACAAAAATAAAAATGTATTCTTGTGATGAAGTAGATTCGTATGAACTTGTGAAAAAAATGGTAAATAATCTATCAGAAAGTGAGTTTATAACAGCAATAGAAATATCTAGAAAACTAAATATTGAAAAATCAAATGTAAACAAACAGCTGTATAAACTACATAATGACGGTTTTATTTTTATGATAAGATCGAATCCACCTAAATGGTTTAAAAAAAATGGCATCGATAACGATGACAATGAGAATAACGATACGAAAAAACTAAATAAATCATTTTCTGATACAATTCCGTACTATAAAATTGTTTTATGGAAAGAAAAAAATCCTTGTTCTGCGATAAACGAAT

>OL977076.1 Lumpy skin disease virus isolate LSDV/NMG/2020 RNA polymerase subunit 30 kDa (RPO30) gene, complete cds

ATGGATGATGATAATACTAATTCATATAGTGATAATACTACCCCCACATATCAAGACATAGAAGATATAATTTATAAATATGTAAAAGAAAAATCAAAGGTAAAAGAAATATTAAAATGGGCAACAGACAAAGCTTCCAAGTTTTATATAAGAAATATTATTAATACAAAGTCAAATATAGAAGAAACAAAATTTGAACCAAGAAACAACATAGGTATTGAATACTCAAAAGATTCAAAAAATAAATTATCGTATAGAAACAAGCCTTTAATAGAGACAAATAAAGATTATTCTGACATATGTGATCTTATACGTACGACAAATGGAACAGAGAAAGAAATTTTAAGATATATACTTTTTGGAATAAAATGTGTTCAAAAAAATGTAGAATTCAATATAGACAATATTAGAGATATAAACCACGAAGAATATTTTAATGTTTTAGATAAAAAGTATAACCTCCCATGCCCTGAGTGCAAAAGTAAAAACACTATTCCCGTCATGATACAAACAAGAGCAGCAGATGAACCACCATTAGTTATGCATTCTTGTAGAGACTGCAAGAAAAATTTTAAACCTCCAAAGTTTAGAGCTGTAGAAAAATAA

>MN864146.1 Lumpy skin disease virus isolate China/XJ/flies/2019 RNA polymerase subunit (lw036) gene, complete cds

CACCAACCATAGAATAGTATCGAGACATGCTATTTCTAACTTTTAAAAAACATTCATCTAAATCATATTTTTTTTCGTTATAAAACTGTTTAAATAACCTACATGCATAAACAGAAGCTTTTTCAAAGTGATTAGGTGGTGTAATATTTTCCATTTTTGCCATTAATGGAAAAATCTAAATAAAGAACTAAAAATTCATTTTTGTAAATATTGAAATTAAACAACTTCAGTACAAATATGGATGATGATAATACTAATTCATATAGTGATAATACTACCCCCACATATCAAGACATAGAAGATATAATTTATAAATATGTAAAAGAAAAATCAAAGGTAAAAGAAATATTAAAATGGGCAACAGACAAAGCTTCCAAGTTTTATATAAGAAATATTATTAATACAAAGTCAAATATAGAAGAAACAAAATTTGAACCAAGAAACAACATAGGTATTGAATACTCAAAAGATTCAAAAAATAAATTATCGTATAGAAACAAGCCTTTAATAGAGACAAATAAAGATTATTCTGACATATGTGATCTTATACGTACGACAAATGGAACAGAGAAAGAAATTTTAAGATATATACTTTTTGGAATAAAATGTGTTCAAAAAAATGTAGAATTCAATATAGACAATATTAGAGATATAAACCACGAAGAATATTTTAATGTTTTAGATAAAAAGTATAACCTCCCATGCCCTGAGTGCAAAAGTAAAAACACTATTCCCGTCATGATACAAACAAGAGCAGCAGATGAACCACCATTAGTTATGCATTCTTGTAGAGACTGCAAGAAAAATTTTAAACCTCCAAAGTTTAGAGCTGTAGAAAAATAACATAAACTATAAAAATGAAAAAAAAAAATTTTTTAGTTTTAAATACAACAAAAATAAAAATGTATTCTTGTGATGAAGTAGATTCGTATGAACTTGTGAAAAAAATGGTAAATAATCTATCAGAAAGTGAGTTTATAACAGCAATAGAAATATCTAGAAAACTAAATATTGAAAAATCAAATGTAAACAAACAGCTGTATAAACTACATAATGACGGTTTTATTTTTATGATAAGATCGAATCCACCTAAATGGTTTAAAAAAAATGGCATCGATAACGATGACAATGAGAATAACGATACGAAAAAACTAAATAAATCATTTTCTGATACAATTCCGTACTATAAAATTGTTTTATGGAAAGAAAAAAATCCTTGTTCTGCGATAAACGAAT

>MW251476.1 Lumpy skin disease virus RNA polymerase subunit 30 kD (RPO30) gene, complete cds

ATGGATGATGATAATACTAATTCATATAGTGATAATACTACCCCCACATATCAAGACATAGAAGATATAATTTATAAATATGTAAAAGAAAAATCAAAGGTAAAAGAAATATTAAAATGGGCAACAGACAAAGCTTCCAAGTTTTATATAAGAAATATTATTAATACAAAGTCAAATATAGAAGAAACAAAATTTGAACCAAGAAACAACATAGGTATTGAATACTCAAAAGATTCAAAAAATAAATTATCGTATAGAAACAAGCCTTTAATAGAGACAAATAAAGATTATTCTGACATATGTGATCTTATACGTACGACAAATGGAACAGAGAAAGAAATTTTAAGATATATACTTTTTGGAATAAAATGTGTTCAAAAAAATGTAGAATTCAATATAGACAATATTAGAGATATAAACCACGAAGAATATTTTAATGTTTTAGATAAAAAGTATAACCTCCCATGCCCTGAGTGCAAAAGTAAAAACACTATTCCCGTCATGATACAAACAAGAGCAGCAGATGAACCACCATTAGTTATGCATTCTTGTAGAGACTGCAAGAAAAATTTTAAACCTCCAAAGTTTAGAGCTGTAGAAAAATAA

>ON691457.1 Lumpy skin disease virus isolate LSDV/China/SiC01/2021 RNA polymerase subunit 30 kDa gene, complete cds

ATGGATGATGATAATACTAATTCATATAGTGATAATACTACCCCCACATATCAAGACATAGAAGATATAATTTAAAATATGTAAAAGAAAAATCAAAGGTAAAAGAAATATTAAAATGGGCAACAGACAAAGCTTCCAAGTTTTATATAGAAATATTATTAATACAAAGTCAAATATAGAAGAAACAAAATTTGAACCAAGAAACAACATAGGTATTGAATATCAAAAGATTCAAAAAATAAATTATTGTATAGAAACAAGCCTTTAATAGAGACAAATAAAGATTATTTTGACATAGTGATTTTATACGTACGACAAATGGAACAGAGAAAGAAATTTTAAGATATATACTTTTTGGAATAAAATGTGTTCAAAAAAATGTAGAATTCAATATAGACAATATTAGAGATATAAACCACGAAGAATATTTTAATGTTTTAGATAAAAAGTATAACCTCCCATGCCCTGAGTGCAAAAGTAAAAACACTATTCCCGTCATGATACAAACAAGAGCAGCAGATGAACCACCATTAGTTATGCATTCTTGTAGAGACTGCAAGAAAAATTTTAAACCTCCAAAGTTTAGAGCTGTAGAAAAATAA

>OL602179.1 Lumpy skin disease virus strain L1103 RNA polymerase subunit 30 kD (RPO30) gene, complete cds

ATGGATGATGATAATACTAATTCATATAGTGATAATACTACCCCCACATATCAAGACATAGAAGATATAATTTATAAATATGTAAAAGAAAAATCAAAGGTAAAAGAAATATTAAAATGGGCAACAGACAAAGCTTCCAAGTTTTATATAAGAAATATTATTAATACAAAGTCAAATATAGAAGAAACAAAATTTGAACCAAGAAACAACATAGGTATTGAATACTCAAAAGATTCAAAAAATAAATTATCGTATAGAAACAAGCCTTTAATAGAGACAAATAAAGATTATTCTGACATATGTGATCTTATACGTACGACAAATGGAACAGAGAAAGAAATTTTAAGATATATACTTTTTGGAATAAAATGTGTTCAAAAAAATGTAGAATTCAATATAGACAATATTAGAGATATAAACCACGAAGAATATTTTAATGTTTTAGATAAAAAGTATAACCTCCCATGCCCTGAGTGCAAAAGTAAAAACACTATTCCCGTCATGATACAAACAAGAGCAGCAGATGAACCACCATTAGTTATGCATTCTTGTAGAGACTGCAAGAAAAATTTTAAACCTCCAAAGTTTAGAGCTGTAGAAAAATAA

>MT448696.1 Lumpy skin disease virus isolate BAN_Pabna_2019 RNA polymerase subunit 30 kD (RPO30) gene, complete cds

ATGGATGATGATAATACTAATTCATATAGTGATAATACTACCCCCACATATCAAGACATAGAAGATATAATTTATAAATATGTAAAAGAAAAATCAAAGGTAAAAGAAATATTAAAATGGGCAACAGACAAAGCTTCCAAGTTTTATATAAGAAATATTATTAATACAAAGTCAAATATAGAAGAAACAAAATTTGAACCAAGAAACAACATAGGTATTGAATACTCAAAAGATTCAAAAAACAAATTATCGTATAGAAACAAGCCTTTAATAGAGACAAATAAAGATTATCCTGACATATGTGATCTTATACGTACGACAAATGGAACAGAGAAAGAAATTTTAAGATATATACTTTTTGGAATAAAATGTGTTCAAAAAAATGTAGAATTCAATATAGACAATATTAGAGATATAAATCACGAAGAATATTTTAATGTTTTAGATAAAAAGTATAACCTCCCATGCCCTGAGTGTAAAAGTAAAAACACTATTCCCGTCATGATACAAACAAGAGCAGCAGATGAACCACCATTAGTTATGCATTCTTGTAGAGACTGCAAGAAAAATTTTAAACCTCCGAAGTTTAGAGCTGTAGAAAAATAA

>OM802146.1 Lumpy skin disease virus isolate NIB/Dinajpur14/2020 RNA polymerase subunit 30 kDa (RPO30) gene, complete cds

ATGGATGATGATAATACTAATTCATATAGTGATAATACTACCCCCACATATCAAGACATAGAAGATATAATTTATAAATATGTAAAAGAAAAATCAAAGGTAAAAGAAATATTAAAATGGGCAACAGACAAAGCTTCCAAGTTTTATATAAGAAATATTATTAATACAAAGTCAAATATAGAAGAAACAAAATTTGAACCAAGAAACAACATAGGTATTGAATACTCAAAAGATTCAAAAAACAAATTATCGTATAGAAACAAGCCTTTAATAGAGACAAATAAAGATTATCCTGACATATGTGATCTTATACGTACGACAAATGGAACAGAGAAAGAAATTTTAAGATATATACTTTTTGGAATAAAATGTGTTCAAAAAAATGTAGAATTCAATATAGACAATATTAGAGATATAAATCACGAAGAATATTTTAATGTTTTAGATAAAAAGTATAACCTCCCATGCCCTGAGTGTAAAAGTAAAAACACTATTCCCGTCATGATACAAACAAGAGCAGCAGATGAACCACCATTAGTTATGCATTCTTGTAGAGACTGCAAGAAAAATTTTAAACCTCCGAAGTTTAGAGCTGTAGAAAAATAA

>MW452636.1 Lumpy skin disease virus isolate LSDV/IND/WB/GS2-LT/2019 RNA polymerase subunit 30 kDa (RPO30) gene, complete cds

ATGGATGATGATAATACTAATTCATATAGTGATAATACTACCCCCACATATCAAGACATAGAAGATATAA

TTTATAAATATGTAAAAGAAAAATCAAAGGTAAAAGAAATATTAAAATGGGCAACAGACAAAGCTTCCAA

GTTTTATATAAGAAATATTATTAATACAAAGTCAAATATAGAAGAAACAAAATTTGAACCAAGAAACAAC

ATAGGTATTGAATACTCAAAAGATTCAAAAAACAAATTATCGTATAGAAACAAGCCTTTAATAGAGACAA

ATAAAGATTATCCTGACATATGTGATCTTATACGTACGACAAATGGAACAGAGAAAGAAATTTTAAGATA

TATACTTTTTGGAATAAAATGTGTTCAAAAAAATGTAGAATTCAATATAGACAATATTAGAGATATAAAT

CACGAAGAATATTTTAATGTTTTAGATAAAAAGTATAACCTCCCATGCCCTGAGTGTAAAAGTAAAAACA

CTATTCCCGTCATGATACAAACAAGAGCAGCAGATGAACCACCATTAGTTATGCATTCTTGTAGAGACTG

CAAGAAAAATTTTAAACCTCCGAAGTTTAGAGCTGTAGAAAAATAA

>MW452638.1 Lumpy skin disease virus isolate LSDV/IND/WB/JS10-LT/2019 RNA polymerase subunit 30 kDa (RPO30) gene, complete cds

ATGGATGATGATAATACTAATTCATATAGTGATAATACTACCCCCACATATCAAGACATAGAAGATATAATTTATAAATATGTAAAAGAAAAATCAAAGGTAAAAGAAATATTAAAATGGGCAACAGACAAAGCTTCCAAGTTTTATAAAGAAATATTATTAATACAAAGTCAAATATAGAAGAAACAAAATTTGAACCAAGAAACAACATAGGTATTGAATACTCAAAAGATTCAAAAAACAAATTATCGTATAGAAACAAGCCTTTAATAGAGACAAATAAAGATTATCCTGACATATGTGATCTTATACGTACGACAAATGGAACAGAGAAAGAAATTTTAAGATATATACTTTTTGGAATAAAATGTGTTCAAAAAAATGTAGAATTCAATATAGACAATATTAGAGATATAAATCACGAAGAATATTTTAATGTTTTAGATAAAAAGTATAACCTCCCATGCCCTGAGTGTAAAAGTAAAAACACTATTCCCGTCATGATACAAACAAGAGCAGCAGATGAACCACCATTAGTTATGCATTCTTGTAGAGACTGCAAGAAAAATTTTAAACCTCCGAAGTTTAGAGCTGTAGAAAAATAA

>MT074114.1 Lumpy skin disease virus strain IND/ODI/8PK/2019 RNA polymerase subunit 30 kDa (RPO30) gene, complete cds

ATGGATGATGATAATACTAATTCATATAGTGATAATACTACCCCCACATATCAAGACATAGAAGATATAATTTATAAATATGTAAAAGAAAAATCAAAGGTAAAAGAAATATTAAAATGGGCAACAGACAAAGCTTCCAAGTTTTATATAAGAAATATTATTAATACAAAGTCAAATATAGAAGAAACAAAATTTGAACCAAGAAACAACATAGGTATTGAATACTCAAAAGATTCAAAAAACAAATTATCGTATAGAAACAAGCCTTTAATAGAGACAAATAAAGATTATCCTGACATATGTGATCTTATACGTACGACAAATGGAACAGAGAAAGAAATTTTAAGATATATACTTTTTGGAATAAAATGTGTTCAAAAAAATGTAGAATTCAATATAGACAATATTAGAGATATAAATCACGAAGAATATTTTAATGTTTTAGATAAAAAGTATAACCTCCCATGCCCTGAGTGTAAAAGTAAAAACACTATTCCCGTCATGATACAAACAAGAGCAGCAGATGAACCACCATTAGTTATGCATTCTTGTAGAGACTGCAAGAAAAATTTTAAACCTCCGAAGTTTAGAGCTGTAGAAAAATAA

>OM362844.1 Lumpy skin disease virus isolate LSDV/Chiplum-14/2020 RNA polymerase subunit 30kDa (RPO30) gene, complete cds

ATGGATGATGATAATACTAATTCATATAGTGATAATACTACCCCCACATATCAAGACATAGAAGATATAATTTATAAATATGTAAAAGAAAAATCAAAGGTAAAAGAAATATTAAAATGGGCAACAGACAAAGCTTCCAAGTTTTATATAAGAAATATTATTAATACAAAGTCAAATATAGAAGAAACAAAATTTGAACCAAGAAACAACATAGGTATTGAATACTCAAAAGATTCAAAAAACAAATTATCGTATAGAAACAAGCCTTTAATAGAGACAAATAAAGATTATCCTGACATATGTGATCTTATACGTACGACAAATGGAACAGAGAAAGAAATTTTAAGATATATACTTTTTGGAATAAAATGTGTTCAAAAAAATGTAGAATTCAATATAGACAATATTAGAGATATAAATCACGAAGAATATTTTAATGTTTTAGATAAAAAGTATAACCTCCCATGCCCTGAGTGTAAAAGTAAAAACACTATTCCCGTCATGATACAAACAAGAGCAGCAGATGAACCACCATTAGTTATGCATTCTTGTAGAGACTGCAAGAAAAATTTTAAACCTCCGAAGTTTAGAGCTGTAGAAAAATAA

>OM362835.1 Lumpy skin disease virus isolate LSDV/malligere-MD/2020 RNA polymerase subunit 30kDa (RPO30) gene, complete cds

ATGGATGATGATAATACTAATTCATATAGTGATAATACTACCCCCACATATCAAGACATAGAAGATATAATTTATAAATATGTAAAAGAAAAATCAAAGGTAAAAGAAATATTAAAATGGGCAACAGACAAAGCTTCCAAGTTTTATATAAGAAATATTATTAATACAAAGTCAAATATAGAAGAAACAAAATTTGAACCAAGAAACAACATAGGTATTGAATACTCAAAAGATTCAAAAAACAAATTATCGTATAGAAACAAGCCTTTAATAGAGACAAATAAAGATTATCCTGACATATGTGATCTTATACGTACGACAAATGGAACAGAGAAAGAAATTTTAAGATATATACTTTTTGGAATAAAATGTGTTCAAAAAAATGTAGAATTCAATATAGACAATATTAGAGATATAAATCACGAAGAATATTTTAATGTTTTAGATAAAAAGTATAACCTCCCATGCCCTGAGTGTAAAAGTAAAAACACTATTCCCGTCATGATACAAACAAGAGCAGCAGATGAACCACCATTAGTTATGCATTCTTGTAGAGACTGCAAGAAAAATTTTAAACCTCCGAAGTTTAGAGCTGTAGAAAAATAA

>OQ943603.1 Lumpy skin disease virus isolate 1792022 hypothetical protein (LSDV035) gene, partial cds; RNA polymerase subunit 30 (LSDV036) gene, complete cds; LSDV034-LSDV034 intergenic spacer, complete sequence; and putative PKR inhibitor (LSDV034) gene, partial cds

GTGGTGTATTATTTTCCATTTTTGCATTAATGGAAAAATTTAAATAAAGAACTAAAAATTCATTTTTGTAAATATTGAAATTAAACAACTTCAGTACAAACATGGATGATGATAATACTAATTCATATAGTGATAATACTAACCCCACATATCAAGACATAGAAGATATAATTTATAAATATGTAAAAGAAAAATCAAAGGTAAAAGAAATATTAAAATGGGCAACAGACAAAGCTTCCAAGTTTTATATAAGAAATATTATTAATACAAAGTCAAATATAGAAGAAACAAAATTTGAACCAAGAAACAACATAGGTATTGAATACTCAAAAGATTCAAAAAACAAATTATCGTATAGAAACAAGCCTTTAATAGAGACAAATAAAGATTATTCTGACATATGTGATCTTATACGTACGACAAATGGAACAGAGAAAGAAATTTTAAGATATATACTTTTTGGAATAAAATGTGTTCAAAAAAATGTAGAATTCAATATAGACAATATTAGAGATATAAATCACGAAGAATATTTTAATGTTTTAGATAAAAAGTATAACCTCCCATGCCCTGAGTGTAAAAGTAAAAACACTATTCCCGTCATGATACAAACAAGAGCAGCAGATGAACCACCATTAGTTATGCATTCTTGTAGAGACTGCAAGAAAAATTTTAAACCTCCGAAGTTTAGAGCTGTAGAAAAATAACATAAACTATAAAAATGAAAAAAAAAAAATTTTTTAGTTTTAAATACAACAAAAAATAAAAATGTATTCTTGTGATGAAGTAGATTCGT

>OP903458.1 Lumpy skin disease virus isolate NIVEDI-LSDV- Haveri 1, RNA polymerase subunit 30 kDa (RPO30) gene, complete cds

ATGGATGATGATAATACTAATTCATATAGTGATAATACTACCCCCACATATCAGGACATAGAAGATATAATTTATAAATATGTAAAAGAAAAATCAAAGGTAAAAGAAATATTAAAATGGGCAACAGACAAAGCTTCCAAGTTTTATATAAGAAATATTATTAATACAAAGTCAAATATAGAAGAAACAAAATTTGAACCAAGAAACAACATAGGTATTGAATACTCAAAAGATTCAAAAAACAAATTATCGTATAGAAACAAGCCTTTAATAGAGACAAATAAAGATTATCCTGACATATGTGATCTTATACGTACGACAAATGGAACAGAGAAAGAAATTTTAAGATATATACTTTTTGGAATAAAATGTGTTCAAAAAAATGTAGAATTCAATATAGACAATATTAGAGATATAAATCACGAAGAATATTTTAATGTTTTAGATAAAAAGTATAACCTCCCATGCCCTGAGTGTAAAAGTAAAAACACTATTCCCGTCATGATACAAACAAGAGCAGCAGATGAACCACCATTAGTTATGCATTCTTGTAGAGACTGCAAGAAAAATTTTAAACCTCCGAAGTTTAGAGCTGTAGAAAAATAA

>OQ030223.1 Lumpy skin disease virus isolate LSD/TN/CUL/TVLR/2022 RNA polymerase 30 kD subunit (RPO30) gene, partial cds

ATGGATGATGATAATACTAATTCATATAGTGATAATACTAACCCCACATATCAAGACATAGAAGATATAATTTATAAATATGTAAAAGAAAAATCAAAGGTAAAAGAAATATTAAAATGGGCAACAGACAAAGCTTCCAAGTTTTATATAAGAAATATTATTAATACAAAGTCAAATATAGAAGAAACAAAATTTGAACCAAGAAACAACATAGGTATTGAATACTCAAAAGATTCAAAAAACAAATTATCGTATAGAAACAAGCCTTTAATAGAGACAAATAAAGATTATTCTGACATATGTGATCTTATACGTACGACAAATGGAACAGAGAAAGAAATTTTAAGATATATACTTTTTGGAATAAAATGTGTTCAAAAAAATGTAGAATTCAATATAGACAATATTAGAGATATAAATCACGAAGAATATTTTAATGTTTTAGATAAAAAGTATAACCTCCCATGCCCTGAGTGTAAAAGTAAAAACACTATTCCCGTCATGATACAAACAAGAGCAGCAGATGAACCACCATTAGTTATGCATTCTTGTAGAGACTGCAAGAAAAATTTTAAACCTCCGAAGTTTAGAGCTGTAGAAAAATAA

>OQ030222.1 Lumpy skin disease virus isolate LSD/TN/CUL/Kris/2022 RNA polymerase 30 kD subunit (RPO30) gene, partial cds

ATGGATGATGATAATACTAATTCATATAGTGATAATACTAACCCCACATATCAAGACATAGAAGATATAATTTATAAATATGTAAAAGAAAAATCAAAGGTAAAAGAAATATTAAAATGGGCAACAGACAAAGCTTCCAAGTTTTATATAAGAAATATTATTAATACAAAGTCAAATATAGAAGAAACAAAATTTGAACCAAGAAACAACATAGGTATTGAATACTCAAAAGATTCAAAAAACAAATTATCGTATAGAAACAAGCCTTTAATAGAGACAAATAAAGATTATTCTGACATATGTGATCTTATACGTACGACAAATGGAACAGAGAAAGAAATTTTAAGATATATACTTTTTGGAATAAAATGTGTTCAAAAAAATGTAGAATTCAATATAGACAATATTAGAGATATAAATCACGAAGAATATTTTAATGTTTTAGATAAAAAGTATAACCTCCCATGCCCTGAGTGTAAAAGTAAAAACACTATTCCCGTCATGATACAAACAAGAGCAGCAGATGAACCACCATTAGTTATGCATTCTTGTAGAGACTGCAAGAAAAATTTTAAACCTCCGAAGTTTAGAGCTGTAGAAAAATAA

>OR678467.1 Lumpy skin disease virus isolate KU_LSD_75 RPO30 gene, complete cds

ATGGATGATGATAATACTAATTCATATAGTGATAATACTAACCCCACATATCAAGACATAGAAGATATAATTTATAAATATGTAAAAGAAAAATCAAAGGTAAAAGAAATATTAAAATGGGCAACAGACAAAGCTTCCAAGTTTTATATAAGAAATATTATTAATACAAAGTCAAATATAGAAGAAACAAAATTTGAACCAAGAAACAACATAGGTATTGAATACTCAAAAGATTCAAAAAACAAATTATCGTATAGAAACAAGCCTTTAATAGAGACAAATAAAGATTATTCTGACATATGTGATCTTATACGTACGACAAATGGAACAGAGAAAGAAATTTTAAGATATATACTTTTTGGAATAAAATGTGTTCAAAAAAATGTAGAATTCAATATAGACAATATTAGAGATATAAATCACGAAGAATATTTTAATGTTTTAGATAAAAAGTATAACCTCCCATGCCCTGAGTGTAAAAGTAAAAACACTATTCCCGTCATGATACAAACAAGAGCAGCAGATGAACCACCATTAGTTATGCATTCTTGTAGAGACTGCAAGAAAAATTGTAAACCTCCGAAGTTTAGAGCTGTAGAAAAATAA

>OR689755.1 Lumpy skin disease virus isolate KU_LSD_130 RPO30 gene, partial cds

ATGGATGATGATAATACTAATTCATATAGTGATAATACTAACCCCACATATCAAGACATAGAAGATATAATTTATAAATATGTAAAAGAAAAATCAAAGGTAAAAGAAATATTAAAATGGGCAACAGACAAAGCTTCCAAGTTTTATATAAGAAATATTATTAATACAAAGTCAAATATAGAAGAAACAAAATTTGAACCAAGAAACAACATAGGTATTGAATACTCAAAAGATTCAAAAAACAAATTATCGTATAGAAACAAGCCTTTAATAGAGACAAATAAAGATTATTCTGACATATGTGATCTTATACGTACGACAAATGGAACAGAGAAAGAAATTTTAAGATATATACTTTTTGGAATAAAATGTGTTCAAAAAAATGTAGAATTCAATATAGACAATATTAGAGATATAAATCACGAAGAATATTTTAATGTTTTAGATAAAAAGTATAACCTCCCATGCCCTGAGTGTAAAAGTAAAAACACTATTCCCGTCATGATACAAACAAGAGCAGCAGATGAACCACCATTAGTTATGCATTCTTGTAGAGACTGCAAGAAAAATTGTAAACCTCCGAAGTTTAGAGCTGTAGAAAAA
