# Supplementary material for: Retrospective analysis of genetic diversity, clinical manifestations and risk factors, during outbreak of lumpy skin disease virus
Source: PLoS One. 2026 Oct 16;21(10):e0358559. doi: 10.1371/journal.pone.0358559 (PMC13674707; doi:10.1371/journal.pone.0358559)
Supplement: Supplementary file 3 [file pone.0358559.s001.doc]

**The Islamia University of Bahawalpur, Bahawalpur, Pakistan.**

**CONSENT STATEMENT**

Dear sir I am Ph.D Scholar in The Islamia University of Bahawalpur, Bahawalpur, Pakistan. These samples and questionnaire are part of my research work. I would greatly appreciate your help in sampling and filling out this questionnaire for my research project. As this is projected, I ask you to please fill it properly and all information obtained will be kept completely confidential and will be used for statistical and analytical purposes only the necessary data will be published for the welfare of human beings and for the novel methodology as can be applicable in Pakistan.

Thank you.

**QUESTIONNAIRE**

Name: _____________________________	Father Name: _______________________________

Address: _________________________________________________________________________ _________________________________________________________________________________

Date of Collection: ___________________	Occupation: ______________________________

**Basic Demographic Features**

**Age (years)	Sex**

| Male |  | Female |  |
| --- | --- | --- | --- |

Education

| Illiterate |  | Read/write |  | Primary |  | Secondary |  | Grade 10. |  |
| --- | --- | --- | --- | --- | --- | --- | --- | --- | --- |
| Intermediate |  | Graduate |  | Postgraduate |  |  |  |  |  |

Income

| 3000 |  | 3000–5000 |  | 5000–10000 |  | 10000–20000 |  | 20000–350000 |  | >35000 |  |
| --- | --- | --- | --- | --- | --- | --- | --- | --- | --- | --- | --- |

**Domestic animals in house**

| **Yes** |  | **No** |  |
| --- | --- | --- | --- |

**If yes, the what type of animals are there in your house**

**Domestic animals in house**

| **Cattles** |  | **Dogs** |  |
| --- | --- | --- | --- |

**Patient in house**

| Yes | No |
| --- | --- |

**Date of onset of symptoms**

**Coordinates of patient household**

**Slaughtering of Diseased Animals**

| Yes | No |
| --- | --- |

**Consumption of diseased animal meat**

| Yes | No |
| --- | --- |

**Disposal of suspected Carcass**

| Yes | No |
| --- | --- |

**Profession of the Patient**

| Farm owner |  | Farm worker |  | Butcher/ Meat Seller |  | Veterinarians |  |
| --- | --- | --- | --- | --- | --- | --- | --- |

**If Butcher/ Meat seller**

| Cattles |  | Sheep/Goat |  |
| --- | --- | --- | --- |

**Type of animals for slaughtering?**

**Previously, butcher have any cut on the skin?**

| Yes | No |
| --- | --- |

**Use of Gloves during slaughtering or meat sell?**

| Yes | No |
| --- | --- |

**Use of spray and proper treatment during the epidemic condition**

**Use of meat of the infected cattle**

| Yes | No |
| --- | --- |

**Site of Lesion**

**Antibiotic Therapy**

| Yes | No |
| --- | --- |

Write the full background of your family

| Separate |  | Joint |  | Male total |  | Female Total |  | Large |  | Small |  |
| --- | --- | --- | --- | --- | --- | --- | --- | --- | --- | --- | --- |

**Population**

| Urban |  | Rural |  | Migrated |  |
| --- | --- | --- | --- | --- | --- |

**Blood serology tests done**

| Yes | No |
| --- | --- |

**Date of outbreaks**

**Test of soil, food water**

| Yes | No |
| --- | --- |

**Stagnant water near the house**

| Yes | No |
| --- | --- |

Any Additional Findings/ Comments:

__________________________________________________________

__________________________________________________________

__________________________________________________________

__________________________________________________________

__________________________________________________________

__________________________________________________________

__________________________________________________________

Signature

_____________________
